# Structure-aware deep learning enhances m6A prediction and reveals cell type-associated RNA structural signatures

**DOI:** 10.1371/journal.pcbi.1014649

**Published:** 2026-08-14

**Authors:** Mingze Sun, Di Zhang, Zhiyuan Li, Yihan Lin

**Affiliations:** 1 Yingcai Honors College, University of Electronic Science and Technology of China, Chengdu, Sichuan, China; 2 Peking University Chengdu Academy for Advanced Interdisciplinary Biotechnologies, Chengdu, Sichuan, China; 3 Center for Quantitative Biology and Peking-Tsinghua Center for Life Sciences, Academy for Advanced Interdisciplinary Studies, Peking University, Beijing, China; 4 The MOE Key Laboratory of Cell Proliferation and Differentiation, School of Life Sciences, Peking University, Beijing, China; Clemson University, UNITED STATES OF AMERICA

## Abstract

N6-methyladenosine (m6A), the most abundant mRNA modification in eukaryotes, plays essential roles in gene regulation and disease pathogenesis. Computational prediction of m6A sites offers a scalable alternative to costly experimental approaches, yet current methods rely predominantly on linear sequence features. This overlooks potentially informative RNA structural context, which is associated with local methylation patterns and may provide complementary predictive information beyond linear sequence motifs. To incorporate this complementary information, we propose SMART-m6A (**S**equence–structure **M**ultifeature **A**ttention **R**NA **T**ransformer for m6A), a deep learning framework that integrates sequence and structural information through parallel convolutional feature extraction and structure-guided attention for multifeature fusion. SMART-m6A achieves superior predictive performance compared to existing methods, with particularly clear advantages in sequence-ambiguous candidates. Beyond prediction accuracy, learned attention patterns reveal strong concordance with experimentally validated m6A-binding protein recognition sites and identify potentially novel regulatory motifs. Through systematic ablation studies and targeted structural-input perturbation analyses, we show that sequence and structure provide complementary predictive information, and that sites with greater prediction sensitivity to structural perturbation exhibit distinct local structural profiles between cell lines. Collectively, this work demonstrates the predictive value of sequence-derived structural features in m6A modeling and provides a multifeature deep learning framework for accurate and interpretable structure-aware epitranscriptomic prediction.

## Author summary

Chemical modifications of RNA provide a crucial regulatory layer beyond the genetic code. Among them, N6-methyladenosine (m6A) is the most abundant internal modification in eukaryotic messenger RNA and has been implicated in diverse biological processes, including development, immune regulation, and tumorigenesis. Understanding how m6A is deposited across transcripts is therefore essential for deciphering epitranscriptomic regulation and its roles in health and disease. Although high-throughput technologies can map m6A sites genome-wide, these experiments remain expensive and technically demanding, motivating the development of computational prediction methods. However, most existing algorithms rely primarily on linear RNA sequence features and largely ignore RNA secondary structure, which has been associated with RNA-binding protein recognition and m6A-related regulatory contexts. Here, we introduce SMART-m6A, a structure-aware deep learning framework that integrates RNA sequence and secondary structure through a multifeature neural architecture with structure-guided attention. By jointly modeling these complementary sources of information, SMART-m6A achieves improved predictive accuracy across multiple benchmark datasets. Importantly, the model is not only predictive but also biologically informative: it identifies structural features associated with m6A deposition, reveals that cell type–specific m6A sites exhibit greater structural variability, and uncovers sequence motifs linked to known RNA-binding proteins. Together, our results indicate that sequence-derived structural features are informative for m6A prediction and highlight the value of structure-aware machine learning approaches for understanding epitranscriptomic mechanisms.

## Introduction

Chemical modifications of RNA represent a crucial layer of post-transcriptional regulation, with more than 160 distinct types identified to date [1–3]. Among them, N6-methyladenosine (m6A) is a modification of mammalian mRNA with essential biological functions, playing pivotal roles in post-transcriptional control [4–9]. The deposition of m6A is dynamically reversible [10]: it is installed by the methyltransferase complex (“writers,” e.g., METTL3/METTL14 [11]/WTAP [12]), removed by demethylases (“erasers,” e.g., FTO [5] and ALKBH5 [13]), and interpreted by a variety of binding proteins (“readers,” including YTH family proteins [14] and hnRNPs [15]). Through these effectors, m6A influences gene expression across multiple steps from transcription to translation, including splicing, nuclear export, translation, and RNA stability [16]. Functionally, m6A serves as a key regulatory node in diverse biological processes and disease phenotypes, such as embryonic development, immune regulation, and tumorigenesis [17]. Advances in next-generation sequencing (NGS) technologies have enabled transcriptome-wide detection of m6A through multiple approaches, including antibody-based methods such as MeRIP-seq [18] and miCLIP [19], and enzyme- or chemistry-based strategies such as MAZTER-seq [20] and m6A-SEAL [21]. In addition, third-generation sequencing methods, such as Nanopore direct RNA sequencing, allow the direct identification of RNA modifications from electrical current signals [22]. More recently, Sun *et al.* introduced the GLORI method, which integrates mild chemical reactions with NGS readouts to achieve absolute quantification of m6A under low-input conditions, thereby opening new possibilities for studies in scarce samples [23]. Despite these advances, the widespread application of m6A profiling remains constrained by the high cost and technical complexity of existing methods.

Meanwhile, computational prediction methods have increasingly emerged as a valuable complement to experimental detection, enabling large-scale predictions within a short timeframe. Early approaches were largely based on traditional machine learning [24]; however, given the intricate interactions within RNA sequences, handcrafted features may not fully capture the underlying rules, which can limit their robustness across datasets. With the advent of deep learning, a growing number of powerful neural architectures have been developed, providing effective tools for modeling RNA sequences. For example, Zhang et al. proposed the DeepM6ASeq framework, which integrates CNNs [25] with BiLSTMs [26] to simultaneously capture local sequence motifs and contextual dependencies [27]. Xiong et al. developed the MASS framework, leveraging multitask learning and attention mechanisms to enable cross-species extraction and interpretation of m6A-related features [28]. Luo et al. introduced iM6A, which incorporates a ResNet [29] architecture to enhance feature representation [30]. More recently, Fan et al. presented deepSRAMP, which combines Transformer [31] and BiGRU [32] modules while integrating genomic positional information, thereby improving m6A prediction accuracy at the isoform level [33]. While these methods have significantly improved m6A prediction accuracy, current approaches face complementary challenges: sequence-only models may lack transcript-level regulatory context, whereas model incorporating positional distribution features, though effective on similar datasets, may inadvertently encode population-level statistics rather than causal sequence properties, potentially limiting robust generalization to novel transcripts or altered cellular contexts.

Recent studies indicate that m6A deposition is not solely determined by canonical sequence motifs such as DRACH [34], but is intricately modulated by the mutual influence between RNA secondary structure and m6A modification itself, likely through the regulation of RNA-binding protein accessibility and binding affinity [35,36]. Shachar et al. demonstrated in vitro that when RNA adopts a stable double-stranded conformation, the METTL3–METTL14 writer complex has limited accessibility for methylation, whereas relaxed single-stranded regions are more readily modified [37]. Wang et al. also reported that Pol II pausing can shape RNA secondary structure, thereby modulating the accessibility of m6A motifs to the methyltransferase complex and influencing subsequent methylation events [38]. Conversely, the m6A-switch model shows that m6A itself can remodel local RNA structure and alter the accessibility of RNA-binding protein motifs, providing evidence that m6A modification can also act upstream of local structural changes [39]. In addition, De La Cruz et al. showed that structurally flexible regions not only facilitate entry of writer complexes (such as METTL3–METTL14–WTAP) but also enhance the binding of reader proteins including YTHDF1/2/3 and regulatory RBPs such as IGF2 BPs and HuD [36]. Collectively, these findings suggest that sequence information alone is insufficient to fully explain the distribution of m6A. Incorporating RNA structural context into computational models may therefore improve m6A prediction and facilitate the identification of structure-associated patterns potentially related to RNA–protein interactions.

Here, we present SMART-m6A, a multifeature deep learning framework that explicitly integrates RNA secondary structure to advance m6A site prediction and uncover structure-associated regulatory signatures in epitranscriptomic patterns. By combining parallel convolutional feature extraction with structure-guided attention, SMART-m6A achieves consistently strong performance across multiple benchmarks and remains applicable when genomic positional annotations are unavailable, addressing a practical limitation in cross-transcript or less-annotated settings. Model interpretability reveals learned attention patterns that recapitulate experimentally validated m6A motifs and identify novel structural signatures enriched at modification sites. Comparative analysis of HEK293T and HeLa m6A landscapes further shows that cell line-specific sites exhibit greater local structural divergence than sites shared between the two cell lines. Model-based structural-input perturbation analysis identifies m6A candidates with differential prediction sensitivity to local structural changes, with prediction-sensitive sites exhibiting greater structural variation in matched icSHAPE profiles. Together with the successful extension to quantitative modification-intensity prediction, these findings support the predictive and interpretive value of sequence-derived structural features and position SMART-m6A as a structure-aware framework for modeling m6A-associated patterns across cellular contexts.

## Results

### Overview of SMART-m6A

SMART-m6A enables joint modeling of RNA sequences and secondary structures, combined with a two-stage training strategy to enhance predictive performance and generalization. The model first employs a dual-branch convolutional module to capture sequence-level k-mer patterns and local structural neighborhood features, which are then integrated within the StructureTransformer encoder. Here, the attention mechanism learns long-range dependencies while explicitly incorporating structural biases, ensuring that global representations retain essential structural constraints. Through the pretraining–supervised learning paradigm, SMART-m6A not only outperforms existing methods across multiple benchmark datasets with accurate site-level identification, but also models the coupling between m6A and secondary structure across cell lines.

### SMART-m6A accurately predicts m6A sites

We trained and evaluated SMART-m6A across six benchmark datasets, including four publicly available antibody-based datasets (SRAMP, DeepM6ASeq, m6ATCPred-Cerebellum7, and m6ATCPred-Lung4) and two datasets constructed in this study from publicly available GLORI-derived data (SMART-m6A and SMART-m6A-Position). Antibody-dependent detection methods are limited in site-level resolution and quantitative precision, whereas GLORI enables single-nucleotide resolution, bias-free, and absolute quantification of m6A via chemical reactions. Evaluating SMART-m6A on such high-quality data provides a stringent test of its predictive power and extends its potential applications to quantitative m6A analyses.

**Fig 1:**
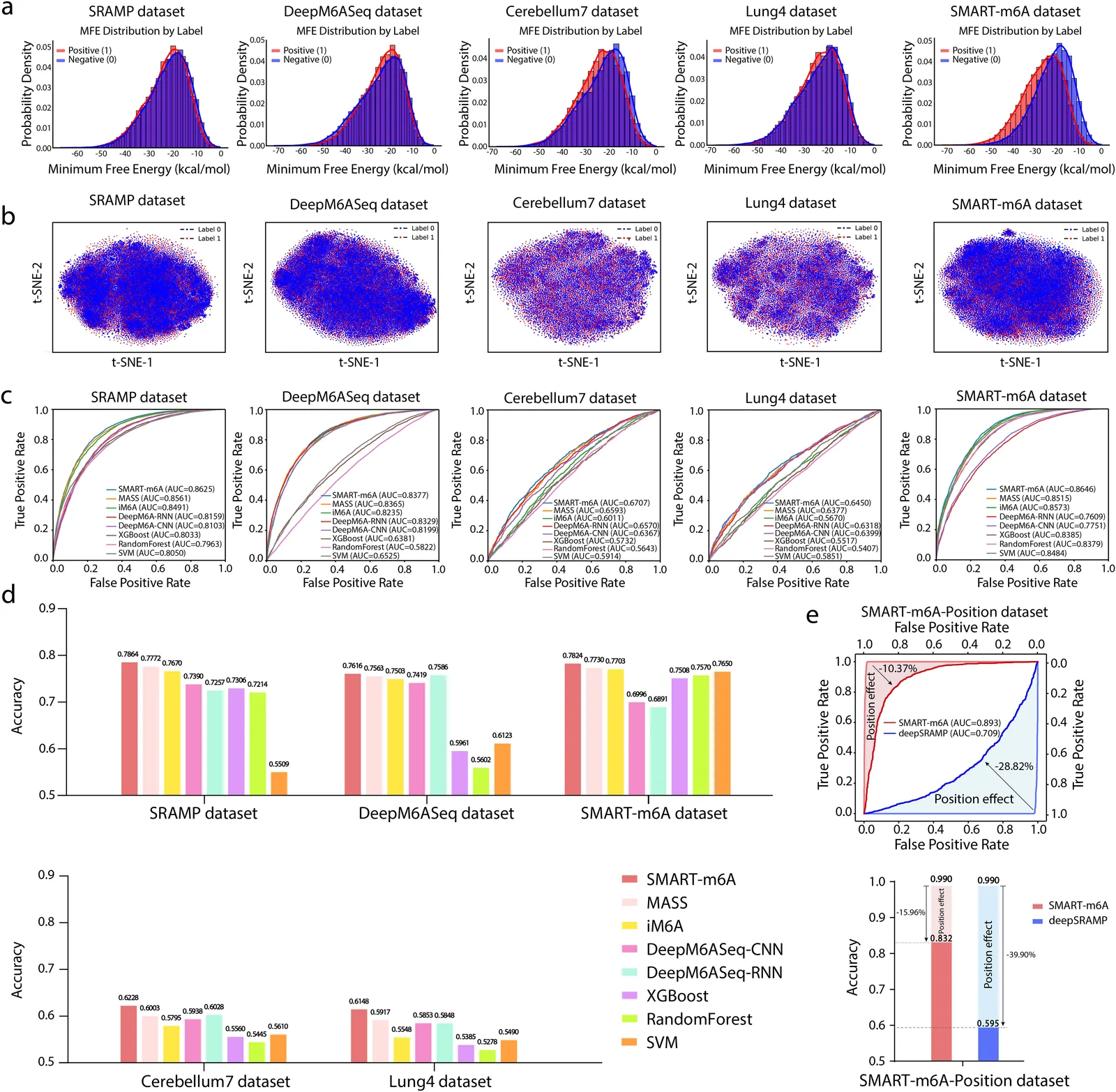
SMART-m6A leverages structural features and achieves superior performance across multiple benchmarks. **(a)** Distribution of minimum free energy (MFE) values calculated by ViennaRNA for positive (with m6A) and negative (without m6A) samples across five benchmark datasets (DeepM6ASeq, SRAMP, m6ATCPred-Cerebellum7, m6ATCPred-Lung4, and SMART-m6A). **(b)** t-SNE visualization of embeddings extracted from the pretrained model across five benchmark datasets. **(c)** ROC curves comparing SMART-m6A against seven representative methods, including MASS, iM6A, DeepM6ASeq-CNN, DeepM6ASeq-RNN, XGBoost, RandomForest, and SVM, on the five benchmark datasets. **(d)** Accuracy comparison of SMART-m6A and baseline models across the five datasets. **(e)** SMART-m6A remains superior to deepSRAMP when positional information is unavailable.

We first performed global feature analyses on five sequence-only datasets, including SRAMP, DeepM6ASeq, m6ATCPred-Cerebellum7, m6ATCPred-Lung4, and SMART-m6A. Minimum free energy (MFE) distributions calculated by ViennaRNA revealed no clear differences between m6A-containing and non-m6A fragments, and the distributional profiles were largely consistent across datasets (Fig 1a). Embeddings extracted from the pretrained model and visualized by t-SNE (Fig 1b) similarly showed no distinct clustering boundaries between positive and negative samples, nor substantial inter-dataset differences. These results indicate that common biophysical metrics or unsupervised learning alone are insufficient to distinguish m6A features, underscoring the necessity of supervised modeling.

To comprehensively assess performance, we compared SMART-m6A against seven representative methods, including four deep learning models (MASS, iM6A, DeepM6ASeq-RNN, and DeepM6ASeq-CNN) and three traditional machine learning models (XGBoost, RandomForest, and SVM). Across all five benchmark datasets, SMART-m6A achieved the best overall performance (Fig 1c and 1d and Tables 1–5). On the GLORI-based SMART-m6A dataset, our model reached an Accuracy of 0.7824, F1-score of 0.7949, AUROC of 0.8646, and AUPRC of 0.8521, outperforming competing approaches. Compared with sequence-only methods, SMART-m6A leveraged secondary structure constraints to resolve sites where sequence context alone was ambiguous, thereby reducing misclassification rates.

**Table 1:** Accuracy of 8 algorithms across 5 datasets.

Dataset	SMART-m6A	MASS	iM6A	DeepM6ASeq-CNN	DeepM6ASeq-RNN	XGBoost	RandomForest	SVM
SRAMP	**0.7864**	0.7772	0.7670	0.7390	0.7257	0.7306	0.7214	0.5509
DeepM6ASeq	**0.7616**	0.7563	0.7503	0.7419	0.7586	0.5961	0.5602	0.6123
Cerebellum7	**0.6228**	0.6003	0.5795	0.5938	0.6028	0.5560	0.5445	0.5610
Lung4	**0.6148**	0.5917	0.5548	0.5853	0.5848	0.5385	0.5278	0.5490
SMART-m6A	**0.7824**	0.7730	0.7703	0.6996	0.6891	0.7508	0.7570	0.7650

**Table 2:** Recall of 8 algorithms across 5 datasets.

Dataset	SMART-m6A	MASS	iM6A	DeepM6ASeq-CNN	DeepM6ASeq-RNN	XGBoost	RandomForest	SVM
SRAMP	0.8124	0.8352	**0.8419**	0.7487	0.6511	0.7436	0.7523	0.1062
DeepM6ASeq	**0.7969**	0.7862	0.7785	0.6984	0.7975	0.6000	0.5860	0.6421
Cerebellum7	0.5745	0.4811	0.7175	**0.7795**	0.7525	0.5550	0.5870	0.5945
Lung4	0.5785	0.6880	0.5425	**0.7455**	0.7335	0.5540	0.5530	0.5990
SMART-m6A	0.8433	**0.8664**	0.7730	0.6955	0.7403	0.7785	0.8062	0.7820

**Table 3:** F1-score of 8 algorithms across 5 datasets.

Dataset	SMART-m6A	MASS	iM6A	DeepM6ASeq-CNN	DeepM6ASeq-RNN	XGBoost	RandomForest	SVM
SRAMP	**0.7918**	0.7894	0.7833	0.7415	0.7036	0.7340	0.7298	0.1912
DeepM6ASeq	**0.7697**	0.7634	0.7572	0.7302	0.7677	0.5977	0.5713	0.6236
Cerebellum7	0.6036	0.5462	0.6305	**0.6574**	0.6545	0.5556	0.5631	0.5752
Lung4	0.6003	0.6276	0.5492	**0.6425**	0.6385	0.5455	0.5394	0.5705
SMART-m6A	**0.7949**	0.7924	0.7709	0.6984	0.7042	0.7575	0.7684	0.7689

**Table 4:** AUROC of 8 algorithms across 5 datasets.

Dataset	SMART-m6A	MASS	iM6A	DeepM6ASeq-CNN	DeepM6ASeq-RNN	XGBoost	RandomForest	SVM
SRAMP	**0.8625**	0.8561	0.8491	0.8103	0.8159	0.8033	0.7963	0.8050
DeepM6ASeq	**0.8377**	0.8365	0.8235	0.8199	0.8329	0.6381	0.5822	0.6525
Cerebellum7	**0.6707**	0.6593	0.6011	0.6367	0.6570	0.5732	0.5643	0.5914
Lung4	**0.6450**	0.6377	0.5670	0.6399	0.6318	0.5517	0.5407	0.5851
SMART-m6A	**0.8646**	0.8515	0.8573	0.7751	0.7609	0.8385	0.8379	0.8484

**Table 5:** AUPRC of 8 algorithms across 5 datasets.

Dataset	SMART-m6A	MASS	iM6A	DeepM6ASeq-CNN	DeepM6ASeq-RNN	XGBoost	RandomForest	SVM
SRAMP	**0.8444**	0.8382	0.8284	0.7774	0.7863	0.7837	0.7845	0.8058
DeepM6ASeq	**0.8251**	0.8243	0.7987	0.8009	0.8201	0.6206	0.5592	0.6219
Cerebellum7	**0.6505**	0.6483	0.5741	0.6202	0.6442	0.5569	0.5455	0.5654
Lung4	**0.6468**	0.6343	0.5558	0.6456	0.6282	0.5487	0.5383	0.5795
SMART-m6A	**0.8521**	0.8259	0.8428	0.7534	0.7401	0.8269	0.8185	0.8272

Previous studies have shown that m6A exhibits non-random transcriptome-wide distribution, typically enriched in the 3′ UTR and near stop codons [2]. Thus, access to genomic positional information can further refine modification predictions. To evaluate this, we benchmarked SMART-m6A against deepSRAMP on the SMART-m6A-Position dataset (Fig 1e and Table 6). When genomic positional information is included, SMART-m6A and deepSRAMP achieve comparable performance. However, in the absence of genomic positional information, both methods exhibit a degree of performance decline, with SMART-m6A showing a much smaller drop than deepSRAMP, demonstrating a clear advantage. Specifically, SMART-m6A experiences a 15.96% reduction in Accuracy (from 0.990 to 0.832) and a 10.34% reduction in AUROC (from 0.996 to 0.893), whereas deepSRAMP suffers a 39.90% reduction in Accuracy (from 0.990 to 0.595) and a 28.82% reduction in AUROC (from 0.996 to 0.709). Even under these conditions, SMART-m6A consistently outperforms deepSRAMP, achieving higher Accuracy (+0.237), Precision (+0.235), F1-score (+0.124), AUROC (+0.183), and AUPRC (+0.145). Considering that genomic positional information is often difficult to obtain in practice, whereas RNA secondary structure features can be computationally inferred from known sequences, the superior performance of SMART-m6A in the no-position setting further highlights its robustness and practical value in real-world applications.

**Table 6:** Performance on the SMART-m6A-Position dataset.

Model	Accuracy	Precision	Recall	F1-score	AUROC	AUPRC
SMART-m6A-Full	0.990	**0.988**	0.995	0.991	0.996	0.995
deepSRAMP-Full	0.990	0.984	**0.999**	0.991	0.996	**0.996**
SMART-m6A	**0.832**	**0.823**	0.900	**0.860**	**0.893**	**0.889**
deepSRAMP-single	0.595	0.588	**0.983**	0.736	0.709	0.744

Table 1–5 Comparison of classification performance metrics (Accuracy, Recall, F1-score, AUROC, AUPRC) of SMART-m6A and baseline models across multiple datasets. Table 6 Comparative performance of SMART-m6A and deepSRAMP variants on the SMART-m6A-Position dataset across multiple evaluation metrics (Accuracy, Precision, Recall, F1-score, AUROC, and AUPRC).

### Ablation experiments support the predictive contribution of structure-derived features

**Fig 2:**
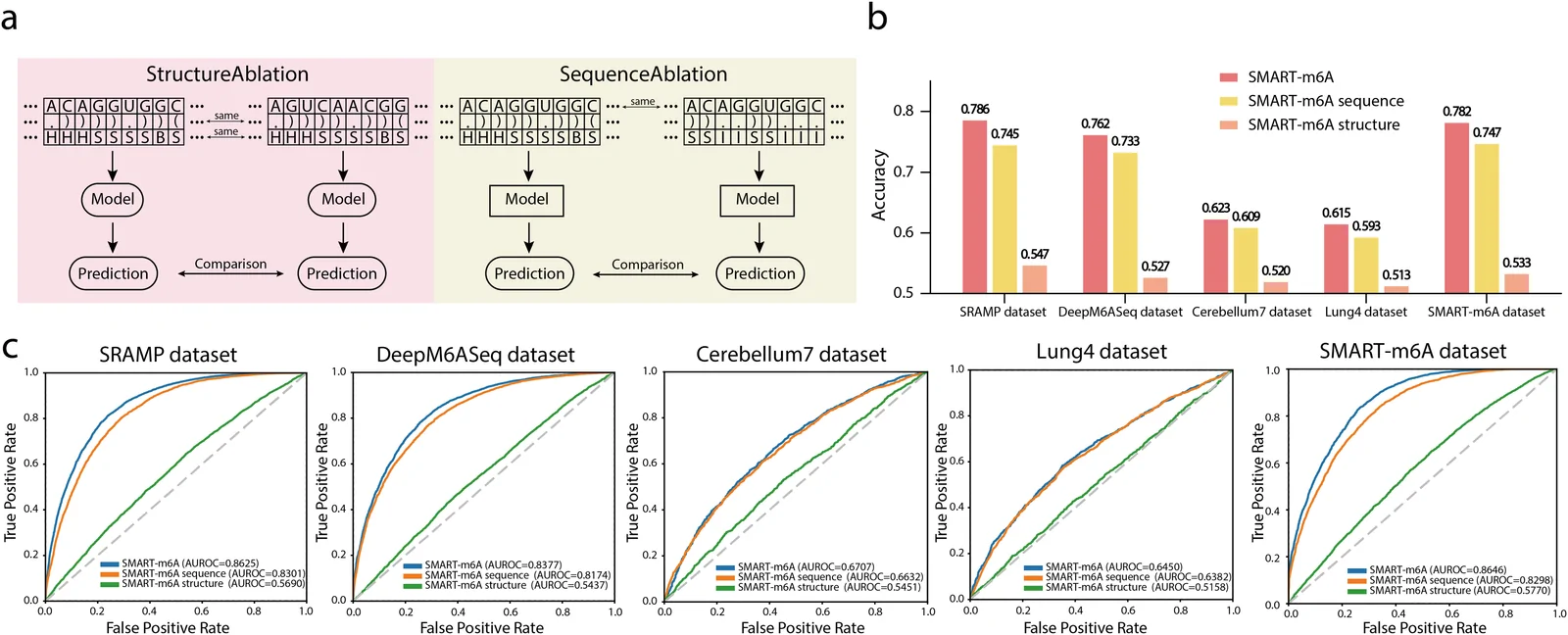
Sequence and structure features provide complementary information for m6A prediction. **(a)** Illustration of ablation settings. In the *StructureAblation* setting, structural features are removed while sequence information is retained; in the *SequenceAblation* setting, sequence features are removed while structural information is retained. Predictions from the ablated models are compared with those of the full SMART-m6A model. **(b)** Accuracy comparison of ablated vs. non-ablated models across five benchmark datasets (SMART-m6A, SRAMP, DeepM6ASeq, m6ATCPred-Cerebellum7, m6ATCPred-Lung4). **(c)** AUROC comparison of ablated vs. non-ablated models across five benchmark datasets.

To systematically assess the relative contributions of sequence and structural features in SMART-m6A, we conducted ablation experiments in two settings: structural ablation and sequence ablation (Fig 2a). In the structural ablation setting, RNA sequences were preserved while loop annotations, dot-bracket structures, and base-pair probability (BPP) matrices were uniformly replaced with those from a fixed reference sample. In the sequence ablation setting, all input sequences were replaced with an identical reference sequence, while structural features were kept intact.

The results revealed that structural ablation led to a modest performance drop, with Accuracy decreasing by 1.4–4.1% and AUROC decreasing by 0.68–3.48% (Fig 2b and 2c). By contrast, sequence ablation caused a substantial degradation in performance, with Accuracy decreasing by 10.2–24.9% to an average of 52.8%, and AUROC decreasing by 12.56–29.40% to an average of 0.5507. As an additional control, we also tested structure-misaligned and random-structure inputs while keeping the sequence inputs and labels unchanged (S1 Fig). Both controls performed similarly to the sequence-only model, whereas the full SMART-m6A model achieved higher ACC and AUROC. These findings indicate that sequence information remains the dominant predictive component, while correctly matched structural features provide complementary information that contributes to the overall predictive performance of SMART-m6A. We reasoned that the contribution of structural features might be underestimated when evaluated on the full test set, because many m6A candidates can be classified primarily from canonical sequence patterns. Therefore, we examined whether structural information provides greater benefit for samples with less confident sequence-only predictions. We defined sequence-only low-confidence samples as the 30% of test samples with the smallest prediction margins under the sequence-only model, representing candidates that were less clearly resolved by sequence information alone. In this subset, the benefit of adding structural features was more pronounced than that observed across all samples (S2 Fig). In the pooled analysis, the Accuracy gain increased from 0.032 in all samples to 0.095 in sequence-only low-confidence samples, and the AUROC gain increased from 0.055 to 0.123. This result indicates that structural features provide clearer additional value for sequence-ambiguous m6A candidates.

### SMART-m6A identifies structurally sensitive m6A sites and models modification levels across cell lines

m6A modifications are reversible and exhibit cell state specificity. Previous studies have reported systematic differences between HeLa and HEK293T m6A landscapes [22]. Given that RNA secondary structures also undergo dynamic changes across cellular contexts and are associated with m6A-related regulatory features, we hypothesized that cell line-specific m6A sites may show more pronounced local structural differences. Moreover, if SMART-m6A indeed captures structure-associated predictive signals, it should be capable of quantifying modification intensities across different cellular states and predicting cell-specific m6A modifications. To test this, we integrated GLORI-based m6A profiles from HeLa and HEK293T cells (including both site locations and modification intensities) with icSHAPE-derived structural data [40] for comparative analysis.

**Fig 3:**
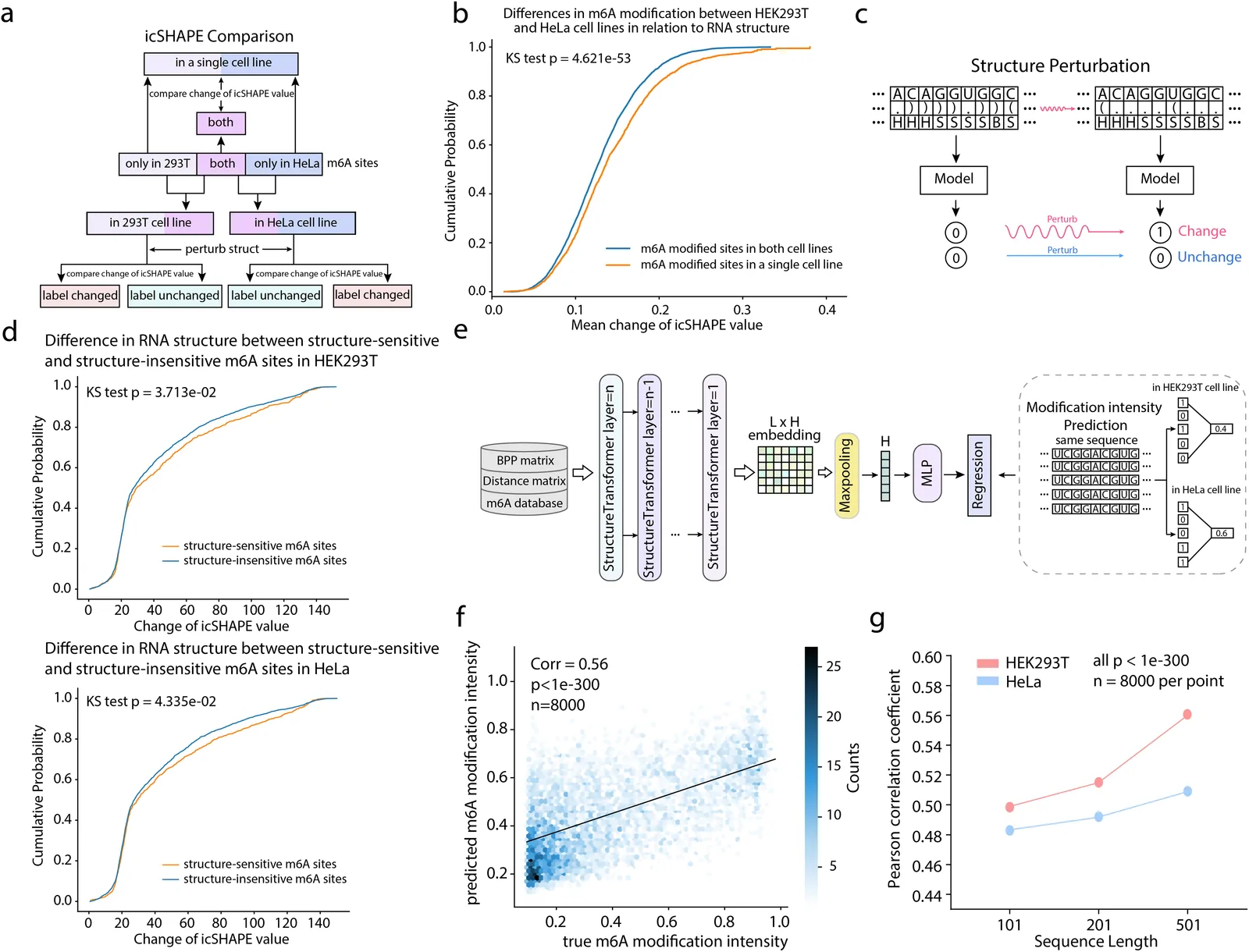
SMART-m6A identifies structure-associated differences in cell line-specific m6A sites and predicts modification intensity. **(a)** Workflow of icSHAPE-based comparison between shared and cell line–specific m6A sites. **(b)** Cumulative distribution of RNA structural probe values for differential versus shared m6A sites between the two cell lines. **(c)** Illustration of the structural perturbation strategy by modifying dot-bracket annotations. **(d)** RNA structural differences between structure-sensitive and structure-insensitive m6A sites in HEK293T and HeLa cells. **(e)** Overview of the regression framework for quantitative prediction of m6A modification intensity. **(f)** Scatter plot showing correlation between predicted and true modification intensity in HEK293T. **(g)** Pearson correlation coefficients under different input sequence lengths (101 nt, 201 nt, 501 nt).

We first summarized the icSHAPE-based comparison workflow (Fig 3a) and quantified cross–cell-line structural divergence between shared and cell line–specific m6A sites using cumulative distributions and a two-sample Kolmogorov–Smirnov (KS) test (Fig 3b). A complementary boxplot comparison is provided in S3 Fig. This analysis revealed a rightward shift for cell line–specific sites, indicating larger icSHAPE changes relative to shared sites (KS, p = 4.621 × 10^-53^). Based on this finding, we further examined whether SMART-m6A could recognize sensitivity to structural input changes. To this end, we designed and implemented a structure-perturbation assay (Fig 3c). Specifically, during the inference stage, we perturbed the 15 consecutive nucleotides with the highest attention scores in each input dot-bracket structural string by swapping the symbols “.” and “()”, and then re-evaluated the model predictions. Sites whose predicted outcomes changed after perturbation were defined as structurally sensitive m6A sites, whereas those remaining unchanged were defined as structurally insensitive m6A sites. We then compared the icSHAPE-based structural variation between these two categories of sites (Fig 3d). The results showed that, in both HEK293T and HeLa cells, structure-sensitive sites exhibited significantly greater structural fluctuations (HEK293T: KS test p = 3.713 × 10^−2^; HeLa: KS test p = 4.335 × 10^−2^). These findings indicate that SMART-m6A can distinguish m6A sites with differential prediction sensitivity to structural perturbation, and that prediction-sensitive sites are associated with greater local structural variation in experimental icSHAPE profiles.

Building on this, we extended SMART-m6A to quantitative prediction of modification intensities (Fig 3e). Considering that the same transcript can exhibit different modification levels at the same adenosine across cell types, we trained models for HeLa and HEK293T separately. As input sequence length increased from 101 nt to 201 nt and 501 nt, correlations between predicted and observed intensities progressively improved (Fig 3f). In HEK293T, the Pearson correlation coefficient reached 0.56, representing a 12.5% increase over the 101-nt model (Fig 3g). In HeLa, correlation reached 0.51, corresponding to a 5.6% increase. Each correlation was calculated using 8,000 paired samples, and all two-sided Pearson correlation tests were highly significant, with $p<\text{1}\times {\text{1}\text{0}}^{-\text{3}\text{0}\text{0}}$. These results demonstrate that longer input sequences provide richer contextual information, thereby enhancing prediction accuracy for modification strength. Moreover, we conducted additional experiments to assess the performance of other algorithms across different sequence lengths (S4 Fig).

Taken together, SMART-m6A captures structure-associated differences in cell line–specific m6A sites and extends to quantitative prediction of modification intensity across cellular contexts. These results highlight the value of integrating structural features for improving model interpretability and for identifying m6A candidates linked to distinct local structural contexts and cell type-dependent modification patterns.

### Motif-based interpretation of SMART-m6A

**Fig 4:**
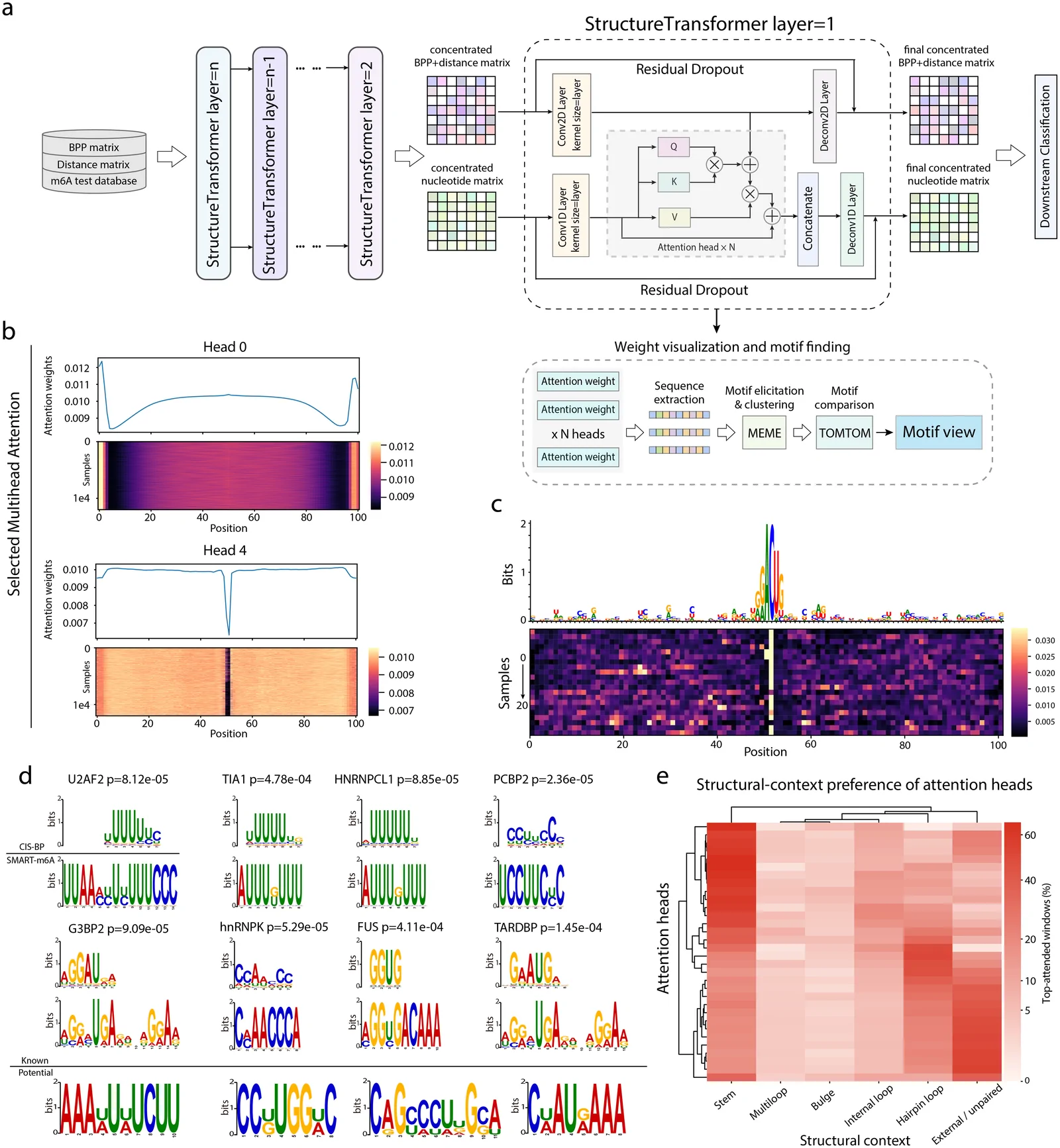
Attention-based interpretability reveals sequence motifs and structural-context preferences associated with m6A prediction. **(a)** Framework of attention weight extraction and motif discovery from the final StructureTransformer layer. **(b)** Global attention distributions across different attention heads. **(c)** Local attention visualization and information content analysis for representative samples. **(d)** Motifs identified from high-attention regions and matched to the CIS-BP human database using MEME–TOMTOM. **(e)** Distribution of top-attended regions across RNA structural contexts for different attention heads.

To explore the interpretability of SMART-m6A, we further analyzed the multi-head attention distribution of the last layer of the StructureTransformer (Fig 4a). Fig 4b presents two representative attention heads, while the results of the remaining heads are provided in S5 Fig. The overall distribution revealed that different attention heads exhibited distinct focus patterns, with each head attending to different sequence regions, suggesting that the multi-head mechanism enables the model to capture information across multiple scales and regions (Fig 4b). Meanwhile, we also performed loop-type statistics on the high-scoring regions attended by different attention heads in the SMART-m6A dataset (S6 Fig). For local attention visualization, we selected a representative attention head and representative positive samples to illustrate sample-to-sample heterogeneity in attention patterns. Information content analysis showed a significant enrichment of DRACH motifs, with the highest bit score at the modification center, consistent with the known sequence conservation of m6A sites. Notably, the attention focus of this head varied across sequences, which aligns with biological intuition: key motifs in different m6A sites may be distributed across different regions. This indicates that the model can capture motif-specific differences among m6A-containing sequences (Fig 4c). To systematically identify motifs learned by the attention mechanism, we applied MEME to high-attention regions in positive test samples, yielding 16 high-scoring motifs. These were compared against the human CIS-BP database using TOMTOM (Fig 4d). Several motifs matched known RNA-binding proteins (RBPs), including hnRNPK, which has been reported to interact with METTL3 as a cofactor to influence m6A deposition and post-transcriptional regulation [41], and FUS, which interacts with m6A-modified RNAs to regulate RNA metabolism and contribute to neurodegenerative disease processes [42]. More matched motifs are provided in S7 Fig.

Given the structure-aware design of SMART-m6A, we further mapped top-attended regions from each attention head to their corresponding RNA structural contexts, including stem, multiloop, bulge, internal loop, hairpin loop, and exterior/unpaired regions (Fig 4e). This analysis revealed distinct structural-context preferences across attention heads, indicating that SMART-m6A attention captures not only sequence motifs but also structure-associated signals relevant to model prediction. To further examine whether these attention-selected structural regions carried predictive information, we extracted structural motif patterns from top-attended windows and compared them with matched background windows (S8a Fig). Several structural motifs were enriched in high-attention regions (S8b Fig). Sequence motifs recovered from windows containing these enriched structural motifs matched DRACH-family m6A motifs, indicating that the enriched structural contexts are coupled with known m6A sequence signals (S8c Fig). In addition, perturbing attention-selected structural windows caused larger prediction changes than perturbing matched high-attention random windows (S8d Fig). These results suggest that the structural regions highlighted by attention are not merely descriptive, but are also functionally relevant to SMART-m6A prediction.

Together, these findings confirm that SMART-m6A successfully captures biologically relevant signals associated with m6A regulation from both sequence and structural contexts. Importantly, we also identified four motifs without significant matches in existing databases. Although their functions remain unclear, these motifs may represent previously uncharacterized regulatory elements, providing potential leads for future experimental validation.

## Discussion

In this study, we developed SMART-m6A, a multifeature deep learning framework that integrates RNA sequence information with sequence-derived structural representations to predict m6A modification sites and modification intensities. Across multiple benchmark datasets, SMART-m6A achieved consistently strong predictive performance, with structural features providing particularly clear additional value for sequence-ambiguous candidates. Attention-based analyses and structural-input perturbation experiments further identified prediction-relevant local sequence–structure patterns. In parallel, cross-cell line analyses showed that cell line-specific m6A sites are associated with greater local structural divergence than shared sites. Together, these results support SMART-m6A as an interpretable structure-aware prediction framework and provide testable hypotheses regarding the relationship between local RNA structural context and cell line-associated m6A patterns.

Despite these advantages, several limitations remain. First, the predictive accuracy of SMART-m6A is inevitably influenced by the quality and resolution of available data. For instance, current icSHAPE profiles and m6A annotations still contain experimental noise and variability, which may constrain the model’s ability to capture subtle local structural features associated with m6A patterns. Second, although our framework effectively integrates sequence and structural features, it does not yet account for additional regulatory factors, such as RNA-binding protein dynamics, cellular stress responses, or epitranscriptomic crosstalk. These factors likely contribute to the cell-specific deposition of m6A. In addition, SMART-m6A uses sequence-derived local structural proxies from isolated mature mRNA fragments, rather than native co-transcriptional RNA structures in vivo.

Looking forward, our results suggest that structure-informed modeling holds strong potential for understanding cell type–specific m6A regulation. Future work could benefit from integrating higher-resolution structural datasets, multi-omics measurements, or single-cell m6A maps to disentangle the interplay between sequence context, secondary structure, and cellular environment. While our study represents a step toward more interpretable and biologically grounded prediction models, it also raises important questions that remain to be addressed, paving the way for deeper exploration of the principles governing epitranscriptomic regulation.

## Materials and methods

### Datasets

We evaluated SMART-m6A across six benchmark datasets, including four publicly available resources (SRAMP [43], DeepM6ASeq [27], m6ATCPred-Cerebellum7 [44], and m6ATCPred-Lung4 [44]) and two in-house datasets (SMART-m6A and SMART-m6A-Position). The dataset sources, detection technologies, training and test sizes, and availability of positional information are summarized in S1 Table. The two datasets constructed in this study were newly generated using the recently developed GLORI technology. We downloaded single-nucleotide resolution m6A modification sites identified using the GLORI method and mapped them to their corresponding genomic coordinates. During dataset construction, negative samples were selected to ensure that the central nucleotide was adenosine (A) and conformed to the DRACH motif, thereby increasing the stringency and difficulty of prediction. All datasets consisted of 101-nt mammalian mRNA fragments. In SRAMP, Cerebellum7, Lung4, and SMART-m6A, sequences were extracted by centering on candidate adenosines, whereas DeepM6ASeq imposed no central nucleotide restriction. Training and test sets were strictly separated for each dataset, and positive and negative samples were approximately balanced in all benchmark datasets. For structural feature data, we used ViennaRNA [45] to generate each sequence’s base-pairing probability matrix and dot-bracket pairing annotation, and employed bpRNA [46] to derive the corresponding loop-domain (loop type) structures. All structural features used in SMART-m6A were computed from isolated mature mRNA fragments. In this setting, the structural inputs represented sequence-derived local structural proxies, and no modification-aware folding model or m6A-aware folding parameters were introduced during model training or cross-validation. In addition, a three-channel relative distance matrix was custom-computed, where each element is inversely proportional to the absolute nucleotide distance $|i-j{|}^{\text{k}}\;$ for three channels (k=1,2,3), with non-zero values only when ∣i−j∣>0. For all datasets, model training and evaluation were conducted under a tenfold cross-validation scheme.

### Dual-branch convolutional feature extraction

**Fig 5:**
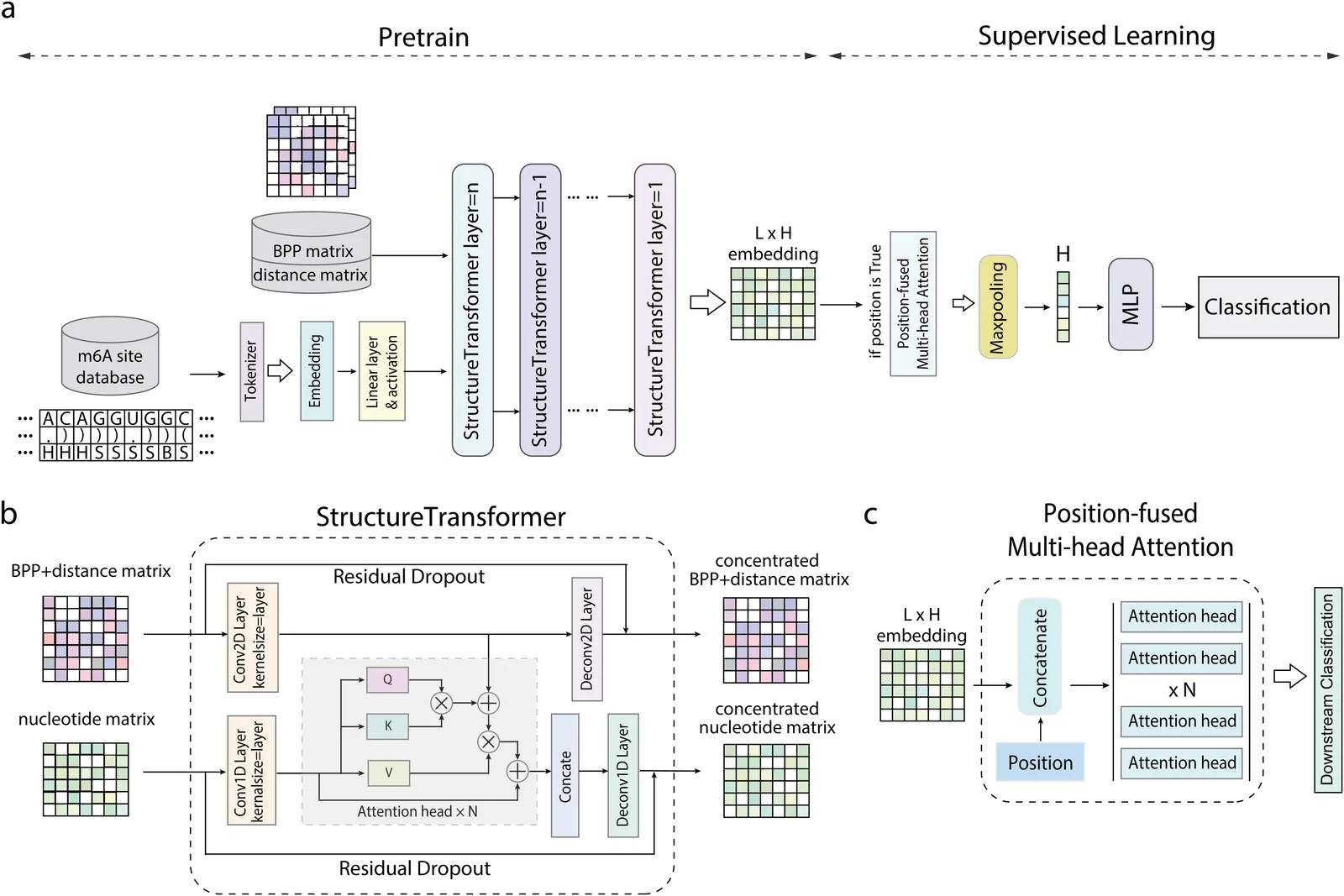
Architecture and workflow of SMART-m6A for multifeature sequence-structure integration. **(a)** Overall workflow of SMART-m6A, from sequence and structure input to m6A site classification. **(b)** StructureTransformer architecture integrating nucleotide and structural features. **(c)** Position-fused multi-head attention incorporating positional information into embeddings.

To jointly capture local sequence patterns and neighborhood structural relationships, SMART-m6A (Fig 5a) employs parallel convolutional modules for the sequence and structural branches at the input stage.

In the sequence branch, each RNA fragment of length L is described by three parallel symbolic channels representing its nucleotide identity (A,C,G,U), dot-bracket structural annotation (paired “()” or unpaired “.”), and loop-context category such as hairpin, internal, bulge, and other types of loops. Each of these channels is first embedded into a dense vector representation through an independent embedding matrix, yielding $E^{\left(\text{seq}\right)}$, $E^{\left(\text{dot}\right)}$, and $E^{\left(\text{loop}\right)}$in $R^{L\times d_{\text{model}}}$. The three embeddings are then concatenated along the last dimension and linearly projected into a unified feature space through a learnable projection matrix $W_{p}\in R^{\text{3}d_{\text{model}}\times d_{\text{model}}}$, forming


$$\begin{array}{c} \begin{array}{c} X=\left[E^{\left(seq\right)}\left|E^{\left(dot\right)}\right|E^{\left(loop\right)}\right]W_{p}. \end{array} \end{array}$$ (1)

Here, [ · ∥ · ] denotes vector concatenation, $d_{\text{model}}$ is the embedding dimension, and the resulting tensor $X\in R^{B\times L\times d_{\text{model}}}$ integrates base-composition and structural semantics for each nucleotide, where B is the batch size.

To capture local contextual dependencies, SMART-m6A applies a one-dimensional convolution across the sequence dimension, such that the output at position p is computed as


$$\begin{array}{c} \begin{array}{c} O_{p}=\sum_{t=\text{0}}^{k-\text{1}}W_{t}\cdot X_{p+t},\;p\in [\text{1},L-k+\text{1}], \end{array} \end{array}$$ (2)

where $W_{t}\in R^{d_{\text{model}}\times d_{\text{model}}}$ denotes the kernel weight at offset t, and $X_{p+t}$ is the projected embedding at position p+t. The resulting vector $O_{p}\in R^{d_{\text{model}}}$ serves as a structure-aware k-mer embedding centered at position p. Owing to the convolution operation, the sequence length is reduced from L to L−k+1, yielding an overall output tensor $O\in R^{B\times (L-k+\text{1})\times d_{\text{model}}}$ that preserves the channel dimension while aggregating local context over k-mer windows. By progressively reducing the kernel size k across convolutional layers, the model first learns short-range conserved motifs in shallow layers and gradually expands its receptive field to capture long-range dependencies in deeper ones.

In parallel, the structural branch processes a four-channel tensor $S_{\text{original}}\in R^{\text{4}\times L\times L}$ that combines the base-pairing probability matrix (BPP) with three relative-distance matrices describing the spatial proximity between nucleotide pairs. This tensor is first projected through a 1×1 convolution and then passed through a series of two-dimensional convolutions to extract local neighborhood correlations, producing


$$\begin{array}{c} \begin{array}{c} S=\text{Conv}\text{2}\text{D}\left(S_{\text{original}}\right). \end{array} \end{array}$$ (3)

The resulting tensor $S\in R^{n_{\text{head}}\times L\times L}$ represents head-specific structural feature maps, where $n_{\text{head}}$ is the number of attention heads. The 2D convolutions slide receptive fields across each L×L structural matrix, capturing spatial dependencies between nucleotide pairs, while transposed convolutions and residual connections refine and stabilize the propagation of structural constraints through deeper layers.

Ultimately, the sequence branch yields structure-aware k-mer representations $O\in R^{B\times (L-k+\text{1})\times d_{\text{model}}}$, and the structural branch produces multi-head spatial feature maps $S\in R^{n_{\text{head}}\times L\times L}$. These complementary feature streams are subsequently fused and fed into the Transformer encoder, enabling integrated modeling of sequence and structural information across multiple spatial scales.

### Transformer encoder with structural integration

Following convolutional feature extraction, SMART-m6A employs a structure-integrated Transformer encoder to jointly model global dependencies and secondary-structure constraints. The dual-branch convolutional backbone together with the structure-aware attention mechanism constitutes what we term the StructureTransformer encoder (Fig 5b).

In this framework, the output tensor from the sequence branch is first linearly projected into the query, key, and value matrices $Q,K,V\in R^{B\times L\times d_{k}}$ through independent learnable transformations:


$$\begin{array}{c} \begin{array}{c} Q=XW_{Q},\;K=XW_{K},\;V=XW_{V}, \end{array} \end{array}$$ (4)

where $W_{Q},W_{K},W_{V}\in R^{d_{model}\times d_{k}}$.

In parallel, the structural branch provides two-dimensional convolutional feature maps $S\in \;R^{n_{head}\times L\times L}$, which serve as head-specific structural biases injected into the attention scoring function. The scaled dot-product attention is defined as:


$$\begin{array}{c} \begin{array}{c} \text{Attention}(Q,K,V,S)=\text{Softmax}\left(\frac{QK^{⊤}}{\sqrt{d_{k}}}+\lambda \;\text{Conv2D}(S)\right)V, \end{array} \end{array}$$ (5)

where $d_{k}$ denotes the dimensionality of the key vectors, and λ is a learnable balancing coefficient controlling the contribution of structure-aware biases relative to sequence-based similarity. Here, Conv2D(S) represents an additional two-dimensional convolution applied to the structural feature maps, which reinforces local base-pair correlations and neighborhood dependencies so that the attention weights are simultaneously constrained by both sequence context and RNA secondary structure.

In implementation, the attention outputs are normalized and refined through residual connections and layer normalization, followed by a feed-forward network. A deconvolutional module is then applied to restore the structural resolution, enabling the stacking of multiple encoder layers. Across successive layers, the model begins with broader convolutional kernels to capture coarse contextual patterns and progressively reduces kernel size to focus on fine-grained local dependencies. At each layer, structure-based biases are explicitly incorporated into attention computation, ensuring that sequence–structure coupling remains consistent and is further reinforced during global representation learning.

### Downstream classification

After five layers of StructureTransformer encoding, the resulting joint sequence–structure representation was used as input to the downstream classification module. For datasets without genomic positional information, the encoded embeddings were directly subjected to max pooling, followed by a multilayer perceptron (MLP) for site prediction. For datasets with positional annotations, we first incorporated a multi-head attention module prior to pooling, enabling effective integration of structural features with positional cues and thereby enhancing the model’s ability to capture position-dependent patterns (Fig 5c). The fused representation was then processed by max pooling and an MLP to generate classification outputs, achieving accurate prediction of m6A modification sites. This design ensures that the model retains flexibility and adaptability across diverse data scenarios.

### Training strategy

During training, SMART-m6A adopted a two-stage pretraining–supervised learning paradigm. In the pretraining stage, we designed reconstruction tasks by randomly masking or mutating nucleotides, structural states (dot-brackets), or loop types. An autoregressive loss was applied to optimize the model, requiring it to accurately recover the original information through softmax prediction. This procedure enabled the model to capture the coupling between RNA sequences and their underlying biophysical properties. In the subsequent supervised learning stage, pretrained weights were loaded on the same dataset, and the model was fine-tuned using available labels. Here, a cross-entropy loss was employed to optimize site-level predictions. The multi-layer embeddings produced by the StructureTransformer were aggregated through global pooling and passed to a multilayer perceptron (MLP), enabling accurate identification of m6A modification sites.

### Quantitative regression of m6A modification intensity

For quantitative prediction of m6A modification intensity, we adapted the downstream prediction module of SMART-m6A from binary classification to regression. The StructureTransformer encoder was kept unchanged, while the final classification layer was replaced with a single-neuron regression head. GLORI-derived modification levels were used as quantitative labels. Because GLORI provides modification stoichiometry as a continuous value, labels were represented on a normalized scale between 0 and 1. The regression model was optimized using mean squared error loss between predicted and observed modification levels. Models were trained separately for HEK293T and HeLa datasets to account for cell line–specific modification profiles. To evaluate whether longer sequence contexts improve quantitative prediction, we trained models using 101-nt, 201-nt, and 501-nt input windows centered on candidate m6A sites. Predictive performance was evaluated using Pearson correlation coefficient between predicted and observed modification levels, with corresponding p-values and sample sizes.

### Identifying prediction sensitivity to structural-input perturbation

Some m6A sites may be closely associated with local RNA structural contexts, where pronounced conformational differences are observed, whereas others appear less coupled to structural variation. We employed a structural perturbation assay to distinguish these two types of sites. In this assay, the nucleotide sequence was kept unchanged while the dot-bracket annotations representing RNA secondary structures were systematically perturbed. For each RNA fragment in the test set, consecutive nucleotides with the highest attention scores from the final layer of the StructureTransformer were identified as structurally critical regions, and their structural states were inverted by swapping paired “()” and unpaired “.” symbols, mimicking the local opening or closing of base-paired segments. Sites whose predicted labels changed after perturbation were defined as structure-sensitive sites, whereas those remaining unchanged were defined as structure-insensitive sites. To verify that SMART-m6A correctly distinguishes between these two categories, we compared icSHAPE-derived structural reactivity changes and found that structure-sensitive sites exhibited significantly greater structural variation than structure-insensitive ones (KS test, p < 0.05). This analysis evaluates the sensitivity of SMART-m6A predictions to local structural-input changes and does not establish a causal effect of RNA structure on m6A deposition.

### Motif extraction for model interpretation

To probe the biological signals captured by the model, we extracted the attention matrices from the final layer of the StructureTransformer and averaged them along the vertical dimension to obtain a base-level attention profile across the input sequence. This distribution reflects the relative contribution of each nucleotide to the overall prediction. We then applied the model to positive samples in the test set and selected subsequences with high attention scores for motif discovery using MEME. The identified motifs were subsequently compared against known databases via TOMTOM. Motifs with successful matches were interpreted as previously established biological signals recognized by the model, whereas unmatched motifs may represent putative novel regulatory elements.

## Supporting information

S1 Fig Structure-misaligned and random-structure control analyses. Comparison of ACC and AUROC among the full SMART-m6A model, sequence plus misaligned structure, sequence plus randomly shuffled structure, and sequence-only SMART-m6A on the SMART-m6A dataset.
(TIF)

S2 Fig Structural-feature gains in all samples and sequence-ambiguous samples. (a) Accuracy gain from structural features in all samples and sequence-ambiguous samples across pooled and individual datasets. (b) AUROC gain from structural features in all samples and sequence-ambiguous samples across pooled and individual datasets.
(TIF)

S3 Fig Local structural divergence of shared and cell line-specific m6A sites. (a) Boxplot comparison of icSHAPE differences between shared and cell line-specific m6A sites. (b) Representative cell line-specific m6A sites with MFE and icSHAPE-guided RNA structures.
(TIF)

S4 Fig Quantitative regression performance across different input sequence lengths. Regression performance of SMART-m6A, Transformer, and CNN using different input sequence lengths in HEK293T and HeLa cell lines.
(TIF)

S5 Fig Attention distributions across all heads in the final StructureTransformer layer. Attention distribution of 32 heads in the last layer of the StructureTransformer on the SMART-m6A test set.
(TIF)

S6 Fig Structural-context composition of top-attended windows. Distribution of loop structure types derived from the top 300 high-attention 16-nt segments in each of the 32 heads of the last StructureTransformer layer on the SMART-m6A test set.
(TIF)

S7 Fig Additional RBP motif matches identified by SMART-m6A. Motifs identified by SMART-m6A that additionally matched entries in the CIS-BP database.
(TIF)

S8 Fig Attention-guided structural motif analysis. (a) Workflow of attention-guided structural motif analysis. (b) Structural motifs enriched in top-attended windows compared with matched background windows, shown as log2 odds ratios. (c) Recovered sequence motifs from enriched structural motifs matched DRACH-family m6A motifs. (d) Perturbation of attention-selected structural motifs caused larger prediction changes than matched random perturbations.
(TIF)

S1 Table Summary of datasets used for model evaluation. Summary of dataset sources, detection technologies, training sizes, test sizes, and availability of positional information for all datasets used in this study.
(XLSX)

S1 File Graphical abstract. (DOCX)
