# Supplementary material for: Structure-aware deep learning enhances m6A prediction and reveals cell type-associated RNA structural signatures
Source: PLoS Comput Biol. 2026 Aug 14;22(8):e1014649. doi: 10.1371/journal.pcbi.1014649 (PMC13502593; doi:10.1371/journal.pcbi.1014649)
Supplement: Supplementary file 1 [file pcbi.1014649.s011.pdf]

Response to reviewers

Dear Reviewers:

We sincerely thank you for your careful evaluation and constructive suggestions. We are encouraged that the reviewers recognized the value of integrating RNA structural information into m6A prediction, as well as the technical design, predictive performance, and practical applicability of SMART-m6A. Guided by your comments, we have added new analyses and carefully revised the manuscript to more clearly present SMART-m6A as a reliable, interpretable, and structure-aware framework for m6A prediction.

The major revisions are summarized as follows:

1. We strengthened the performance evaluation of SMART-m6A by adding cross-dataset comparisons with XGBoost, random-seed stability analyses, and clarification of the fixed model configuration used across benchmark datasets.
2. We added structure-misaligned and random-structure controls, together with analyses of sequence-ambiguous samples, to demonstrate that correctly matched structural features provide complementary predictive value, particularly for challenging candidates.
3. We expanded the analysis of cell line-associated structural differences by integrating GLORI-derived m6A profiles with matched icSHAPE data from HEK293T and HeLa cells, including both statistical comparisons and representative site-level examples.
4. We extended the interpretability analysis from sequence motifs to RNA structural contexts and structural motifs, and added perturbation analyses showing that attention-highlighted structural regions contribute to SMART-m6A prediction.
5. We improved the methodological and conceptual presentation of the study by adding the quantitative regression methods, statistical details, a comprehensive dataset summary table, and more precise terminology describing sequence-derived structural features and structure-associated predictive signals.

The principal changes to the figures, supplementary figures, tables, and methodological descriptions are summarized below:

- Figure 4 has been updated to more clearly present the association between local RNA structural divergence and cell line-specific m6A patterns, together with quantitative modification-intensity prediction and the corresponding statistical information.
- Figure 5e has been added to show the structural-context preferences of different attention heads.
- Figure S1 has been added to compare the full SMART-m6A model with structure-misaligned, random-structure, and sequence-only controls.
- Figure S2 has been added to compare the contribution of structural features in all samples and in sequence-ambiguous samples.

- Figure S3 has been expanded to provide complementary statistical comparison and representative examples of cell type-specific m6A sites with matched structural profiles.
- Figure S4 has been modified to compare quantitative regression performance across different input sequence lengths and baseline models.
- Figures S5–S8 have been expanded to provide additional attention-head visualizations, structural-context analyses, motif comparisons, and structural perturbation results.
- Table S1 has been added to summarize the datasets, experimental sources, detection technologies, sample sizes, and positional-information availability used in this study.
- A new Methods subsection, "Quantitative regression of m6A modification intensity," has been added to describe the quantitative prediction task in detail.
- The Materials and Methods and Discussion sections have been revised to more precisely define the structural inputs as sequence-derived local structural proxies and to clarify the interpretation of structure-associated predictive signals identified by SMART-m6A.

These revisions are incorporated into the revised manuscript and are described in detail in the point-by-point responses below. Changes in the revised manuscript are highlighted in red for ease of reference.

Point-by-point reply:

Reviewer #1:

This manuscript presents SMART-m6A, a deep learning tool that integrates structural features to predict m6A sites and identify cell-type-specific patterns. The premise of incorporating RNA structure into epitranscriptomic prediction is interesting. The insight into the association of RNA structure with m6A can be useful to future biological research some translational applications, such as mRNA therapy. There are several concerns regarding the justification of the model's complexity relative to its performance gains, as well as the interpretation and presentation of the results, that need to be addressed.

We thank the reviewer for the careful evaluation of our manuscript and for recognizing the interest of incorporating RNA structure into epitranscriptomic prediction. These comments helped us refine the rationale, analyses, and presentation of SMART-m6A.

To clarify the relationship between model complexity and performance gain, we added cross-dataset comparisons and stability analyses. SMART-m6A achieved higher overall performance than XGBoost, the strongest traditional machine-learning baseline in our benchmark, and remained stable across random seeds using a fixed model configuration. We also cited recent omics modeling literature to support Transformer-based architectures as an appropriate framework for integrating heterogeneous biological inputs.

In addition, the revised manuscript now presents the structural contribution of SMART-m6A more clearly and with stronger supporting evidence. The added analyses show that structural features provide larger benefit in sequence-ambiguous samples, that cell type-

specific m6A patterns are associated with local structural divergence, and that attention-highlighted structural regions are linked to known m6A sequence signals and prediction-relevant model behavior. Together with the clarified dataset descriptions, added statistical details, toned-down speculative wording, and standardized terminology, these revisions make the sequence-structure integration advantage of SMART-m6A more explicit and present the model as a reliable, interpretable, and structure-aware framework for m6A prediction.

Major Comments

1. The authors construct a complex Transformer-based model to incorporate structural features; however, the performance improvements appear marginal. In Tables 1-5 and Figures 2c-2d, SMART-m6A improves the AUC over XGBoost by only about 3%, with other metrics showing only slight, and sometimes inconsistent, improvements. Given that such small differences can often be attributed to hyperparameter tuning variations rather than fundamental architectural advantages, the necessity of this complex model is questionable.

We thank the reviewer for raising this point, which gives us an opportunity to clarify the necessity and stability of the Transformer-based design in SMART-m6A. To address this concern, we added analyses from four aspects: fixed hyperparameter evaluation, random-seed stability, cross-dataset overall performance, and the significance of sequence–structure integration. Together, these analyses show that SMART-m6A achieves stable improvements across heterogeneous datasets through its structure-aware Transformer design.

First, to address the concern about hyperparameter tuning, we clarified that SMART-m6A was evaluated using the fixed model configuration across all benchmark datasets. No dataset-specific architecture or parameter set was introduced for individual datasets. The baseline methods were also evaluated under consistent settings. Therefore, the observed performance difference reflects the behavior of a fixed model design across heterogeneous benchmarks. This supports that the improvement is not driven by dataset-specific parameter selection.

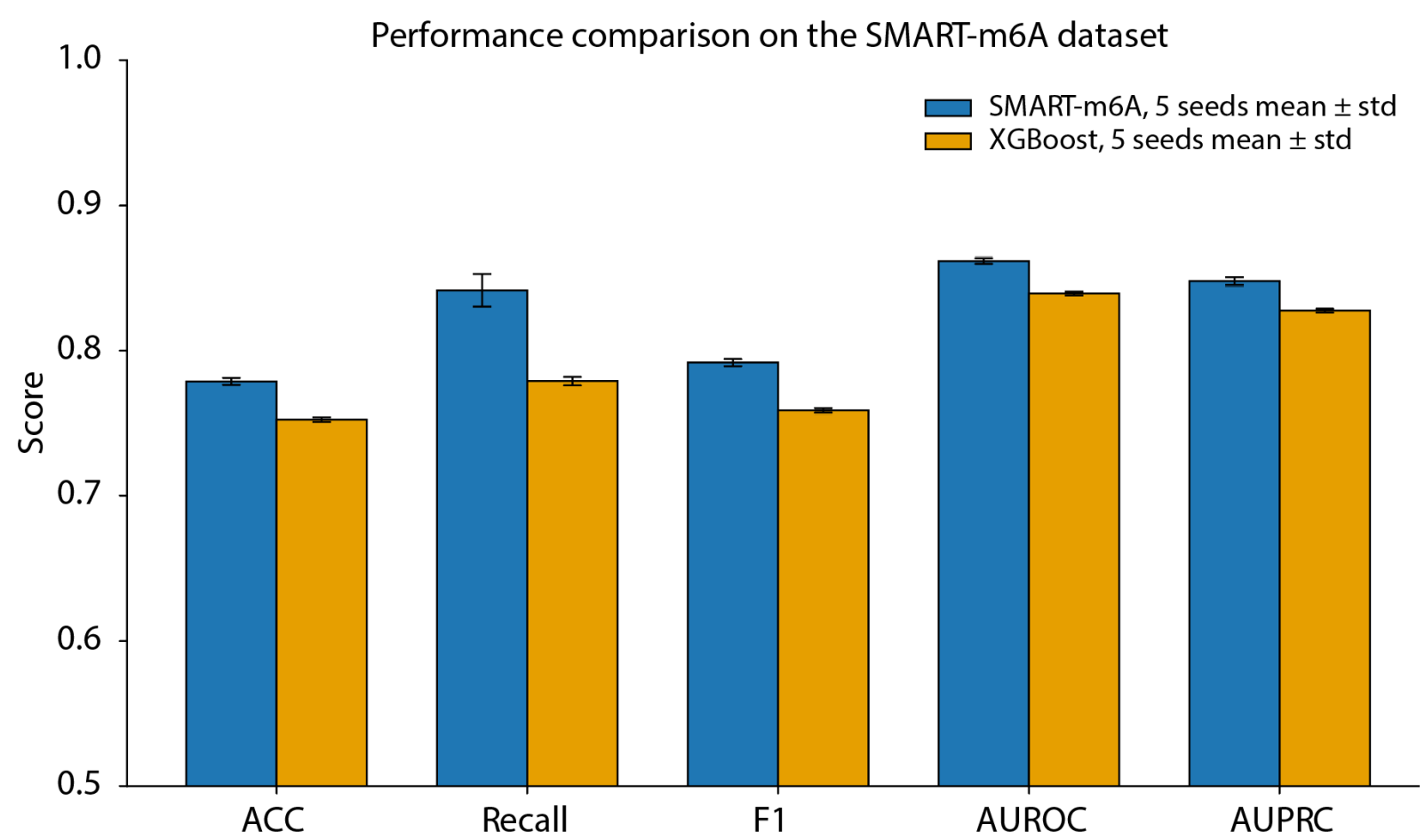

Additional Figure 1. Random-seed stability of SMART-m6A and comparison with XGBoost on the SMART-m6A dataset.

Second, to address the effect of random initialization, we repeated SMART-m6A training and XGBoost on the SMART-m6A dataset using five random seeds. We selected this dataset because XGBoost achieved its strongest performance on this benchmark among the tested datasets, making it a stringent setting for evaluating the stability of SMART-m6A. As shown in Additional Figure 1, SMART-m6A showed small standard deviations across the five runs. These results confirm that SMART-m6A is stable across different random initializations and maintains a consistent performance advantage over XGBoost.

Metric	SMART-m6A mean	XGBoost mean	Absolute gain	Relative gain
Accuracy	0.7136	0.6344	+0.0792	+12.48%
Recall	0.7211	0.6462	+0.0749	+11.59%
F1-score	0.7121	0.6381	+0.0740	+11.60%
AUROC	0.7761	0.6810	+0.0951	+13.97%
AUPRC	0.7638	0.6674	+0.0964	+14.45%

Additional Table 1. Cross-dataset mean performance comparison between SMART-m6A and XGBoost.

Third, to address the overall performance advantage, we added a cross-dataset overall comparison between SMART-m6A and XGBoost. As summarized in Additional Table 1, SMART-m6A achieved higher overall performance than XGBoost across all evaluated metrics. This result shows that the advantage of SMART-m6A is consistent at the cross-dataset level, instead of being restricted to one metric or one benchmark dataset.

Fourth, to address why a Transformer-based model is appropriate for this task, we clarified the motivation of the model architecture. Recent reviews in omics modeling have highlighted Transformer architectures as a major framework for integrating heterogeneous and multimodal biological inputs, because attention mechanisms can model contextual relationships among different input tokens or features (Szałata et al., 2024). However, in m6A site prediction, how to effectively integrate sequence information with RNA structural representations remains less well explored. SMART-m6A was designed to integrate sequence information with multiple sequence-derived structural representations, including base-pairing probability, dot-bracket annotation, loop context, and relative-distance matrices. This design enables SMART-m6A to jointly capture sequence motifs and local structure-associated patterns, making the Transformer-based architecture well suited for sequence–structure integration. Its advantage was most evident on heterogeneous organ-level datasets such as Cerebellum7 and Lung4, and in sequence-ambiguous samples, where structural features produced larger Accuracy and AUROC gains than in the full test set (Figure 2d and Additional Figure 2).

Taken together, we have revised the manuscript to present SMART-m6A as a reliable and interpretable sequence-structure integration framework. The added analyses support that its model design provides consistent predictive gains across datasets, improved robustness across random seeds, and additional value in sequence-ambiguous m6A prediction cases.

References cited in this response:
Szałata, A., Hrovatin, K., Becker, S., Tejada-Lapuerta, A., Cui, H., Wang, B. & Theis, F. J. Transformers in single-cell omics: a review and new perspectives. Nature Methods 21, 1430–1443 (2024). https://doi.org/10.1038/s41592-024-02353-z

2. The authors state that handcrafted features fail to capture underlying rules due to intricate RNA sequence interactions, yet SMART-m6A does not yield a substantial leap in predictive accuracy.

We thank the reviewer for raising this point. First, to address whether SMART-m6A provides a meaningful improvement over handcrafted-feature methods, we added a cross-dataset overall comparison with XGBoost, the strongest traditional machine-learning baseline in our benchmark. As summarized in Additional Table 1, SMART-m6A achieved higher average performance than XGBoost across Accuracy, Recall, F1-score, AUROC, and AUPRC over the five benchmark datasets. This result shows that the improvement of SMART-m6A is not restricted to a small AUROC change on one dataset, but is observed across multiple evaluation metrics at the cross-dataset level.

Second, the dataset-level comparison further supports the robustness of SMART-m6A. As shown in Figure 2d, traditional machine-learning methods showed stronger dataset-dependent variation. For example, XGBoost achieved relatively strong performance on the datasets of SRAMP and SMART-m6A, but its performance decreased on the datasets of DeepM6ASeq, Cerebellum7, and Lung4. Similar fluctuations were also observed for RandomForest and SVM. In contrast, SMART-m6A maintained consistently strong performance across datasets with different sources, construction strategies, and difficulty levels. These results indicate that SMART-m6A provides more stable cross-dataset performance than traditional handcrafted-feature-based methods.

The manuscript has also been refined in the Introduction. Traditional machine-learning methods remain useful, while handcrafted features may have limited capacity to represent broader sequence–structure context across heterogeneous datasets. Therefore, we revised the original wording from handcrafted features “often failed to capture the underlying rules” to “handcrafted features may not fully capture the underlying rules, which can limit their robustness across datasets” (Lines 90–94). The revised manuscript now presents the benchmark results more accurately and frames SMART-m6A as a reliable, broadly applicable, and interpretable framework for sequence-structure integration.

3. In the ablation experiments, removing structural information only decreases accuracy by 1.4–4.1% and AUROC by 0.68–3.48%. Does a performance drop of 1-4% truly indicate that structural information plays a significant enough role to be the primary novel contribution of this paper?

We thank the reviewer for raising this question. The ablation results should be interpreted together with the sample-level difficulty of the prediction task. Many m6A candidates contain strong sequence cues, such as DRACH-like motifs and local nucleotide

composition, and can therefore be classified confidently by sequence information alone. In these cases, adding structural features may improve the prediction score but may not change the final predicted label, which makes the average gain on the full test set appear modest.

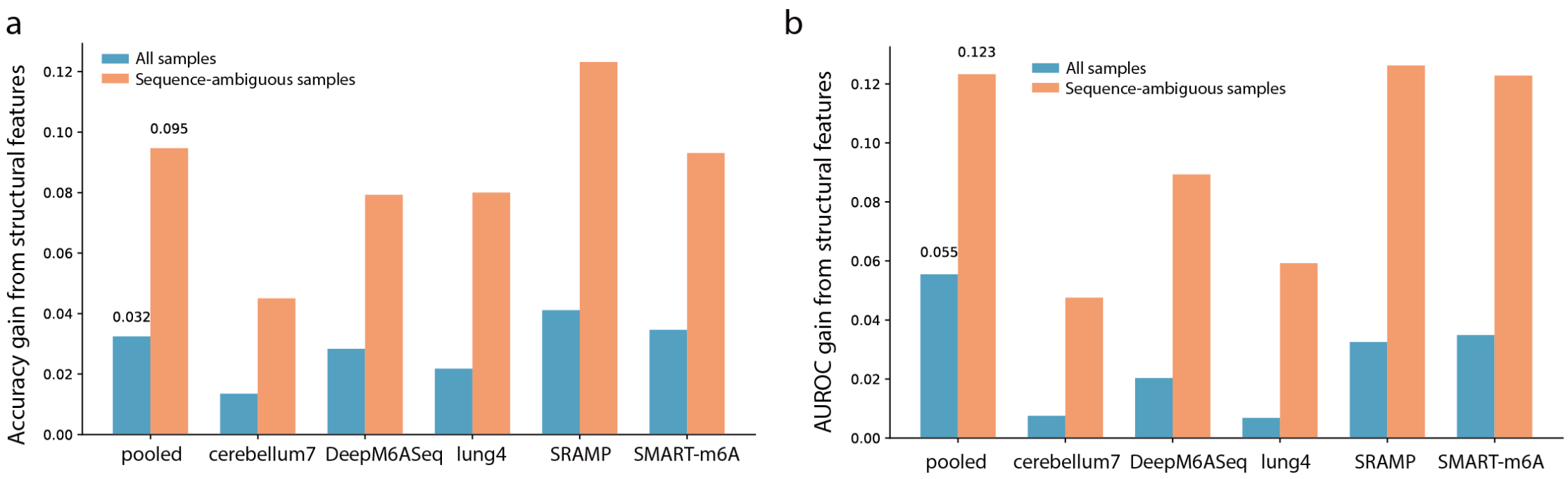

Additional Figure 2. (a) Gain of accuracy from structural features in all samples and sequence-ambiguous samples across pooled and individual datasets. (b) AUROC gain from structural features in all samples and sequence-ambiguous samples across pooled and individual datasets.

To further examine the conditions under which structural features provide the greatest benefit, we performed analyses on a subset of sequence-ambiguous samples. We first identified the 30% of test samples for which the sequence-only model produced predictions closest to the classification boundary. These samples represent candidates that were less clearly resolved by sequence information alone. We then compared the gain from adding structural features in all samples and in this sequence-ambiguous subset. This analysis is now described in Lines 221–230 and shown in Additional Figure 2 (Figure S2). The results show that structural features provide larger gains in sequence-ambiguous samples. In the pooled analysis, the Accuracy gain increased from 3.2% in all samples to 9.5% in sequence-ambiguous samples, and the AUROC gain increased from 5.5% to 12.3%. Similar trends were observed across all datasets. These results indicate that structural features play an important role in sequence-ambiguous samples.

Accordingly, we added this sequence-ambiguous sample comparison to the ablation experiment section, providing a more detailed view of where structural information contributes most clearly. This revision is now described in Lines 221–233 and shown in Figure S2.

4. The authors claim that cell-type-specific m6A patterns are linked to RNA structure. While this is a compelling hypothesis, it currently lacks concrete visual or data-driven examples in the text. To substantiate this claim, please provide specific examples demonstrating the structural differences at these key m6A sites across different cell types.

We thank the reviewer for this suggestion. The relationship between RNA structure and cell type-specific m6A regulation is indeed a key biological issue, and it is also closely related to the concern raised by Reviewer 2. In this context, we would like to clarify that the connection between RNA structure and m6A modification is not a hypothesis introduced uniquely by our study, but is supported by a substantial body of prior work.

Previous icSHAPE-based in vivo RNA structure profiling has shown that RNA structural features can encode regulatory information in living cells and can be used to study RNA regulatory mechanisms (Spitale et al., 2015). Subsequent deep-learning work further demonstrated that in vivo RNA structural profiles can help predict dynamic RNA–protein interactions across cellular conditions (Sun et al., 2021). In the context of m6A, the m6A-switch model showed that m6A can remodel local RNA structure and regulate the accessibility of RNA-binding protein motifs (Liu et al., 2015). Biophysical and structural studies also showed that m6A can alter local RNA duplex stability and stabilize specific junctional RNA motifs in a context-dependent manner (Roost et al., 2015; Liu et al., 2018). Together, these studies indicate a close interplay between m6A modification, local RNA structure, and RNA-protein interaction, although the direction and mechanism of this relationship can be context-dependent.

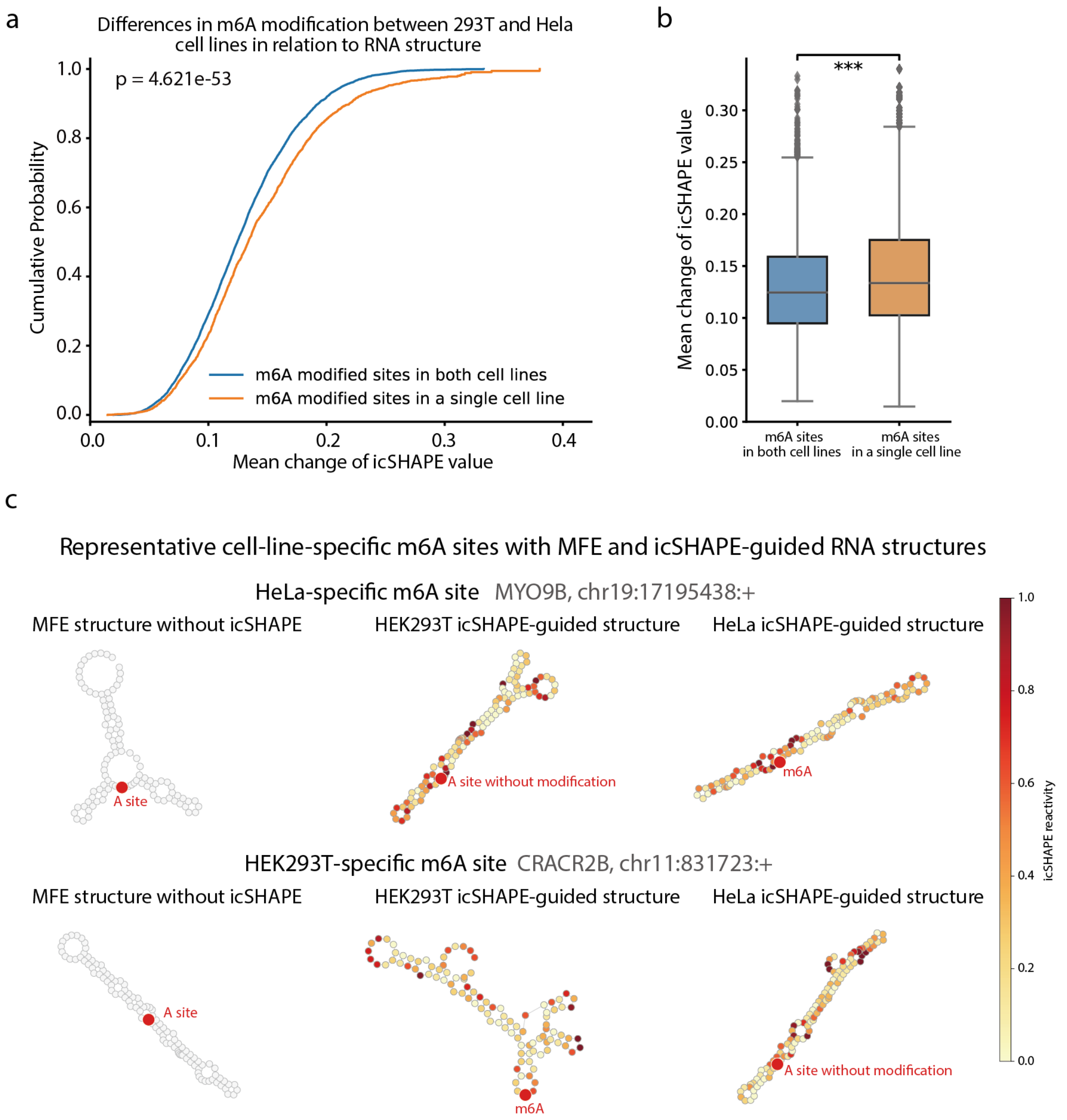

Additional Figure 3. (a) Cumulative distribution of icSHAPE differences for shared and cell line-specific m6A sites. (b) Boxplot comparison of icSHAPE differences between shared and cell line-specific m6A sites. (c) Representative cell-line-specific m6A sites with MFE and icSHAPE-guided RNA structures.

Based on this established biological background, GLORI-derived m6A annotations were integrated with matched icSHAPE profiles from HEK293T and HeLa cells. Shared and cell line-specific m6A sites were then compared using the absolute icSHAPE difference between the two cell types at the same sites. This analysis showed that cell line-specific m6A sites are associated with greater local structural divergence than shared sites. This analysis is now summarized in the revised Results section (Lines 232–249). As shown in Figure 4b (Additional Figure 3a), cell line-specific m6A sites showed a right-shifted distribution of icSHAPE differences compared with m6A sites shared by both cell lines. To provide a more direct visual comparison, we further added a complementary boxplot analysis in Supplementary Figure S3 (Additional Figure 3b), which is now explicitly referenced in the revised manuscript (Line 247). Cell line-specific m6A sites showed higher icSHAPE differences than shared sites. This difference was significant by both the Mann-Whitney U test and the two-sample Kolmogorov-Smirnov test ($p = 4.621e-53$; Lines 244–249). To provide concrete data-driven examples, we added representative cell-line-specific m6A sites with matched MFE and icSHAPE-guided RNA structures from HEK293T and HeLa cells (Additional Figure 3c). For each example, the same local sequence window was folded using the MFE model and further compared with cell-line-specific icSHAPE-guided structures. These examples show that cell-line-specific m6A sites can be associated with visibly different local RNA structural environments between HEK293T and HeLa cells. Together, the population-level statistical analysis and the representative structure example support the association between cell type-specific m6A occurrence and local RNA structural divergence between HEK293T and HeLa.

At the same time, in response to Reviewer 2's concern, the revised manuscript now clarifies that this analysis identifies an association between cell line-specific m6A status and local RNA structural divergence, but does not establish whether structural differences precede or follow m6A modification. The observed differences may reflect structure-associated regulation of m6A deposition, m6A-induced structural remodeling, cell type-specific RBP binding, or a combination of these effects. Accordingly, the revised text describes these results as "structure-associated differences" and "local structural contexts" (Lines 277-281). This icSHAPE analysis therefore supports the biological relevance of sequence-structure information, while SMART-m6A identifies predictive sequence-structure patterns that may guide future experimental validation.

References cited in this response:
Spitale, R. C., Flynn, R. A., Zhang, Q. C., et al. Structural imprints in vivo decode RNA regulatory mechanisms. Nature 519, 486–490 (2015).
Sun, L., Fazal, F. M., Li, P., et al. Predicting dynamic cellular protein–RNA interactions by deep learning using in vivo RNA structures. Cell Research 31, 495–516 (2021).
Liu, N., Dai, Q., Zheng, G., He, C., Parisien, M., & Pan, T. N6-methyladenosine-dependent RNA structural switches regulate RNA–protein interactions. Nature 518, 560–564 (2015).
Roost, C., Lynch, S. R., Batista, P. J., et al. Structure and thermodynamics of N6-methyladenosine in RNA: a spring-loaded base modification. Journal of the American Chemical Society 137, 2107–2115 (2015).
Liu, B., Merriman, D. K., Choi, S. H., et al. A potentially abundant junctional RNA motif stabilized by m6A and Mg2+. Nature Communications 9, 2761 (2018).

5. The study successfully utilizes the attention mechanism for sequence motif analysis. Given the paper's focus on RNA structure, restricting the attention analysis strictly to sequences feels like a missed opportunity. Could the attention mechanism also be applied to structural analysis? It would significantly strengthen the paper if the authors could demonstrate whether the model's attention weights can identify specific structural motifs or conformations that provide physical evidence for the presence of m6A.

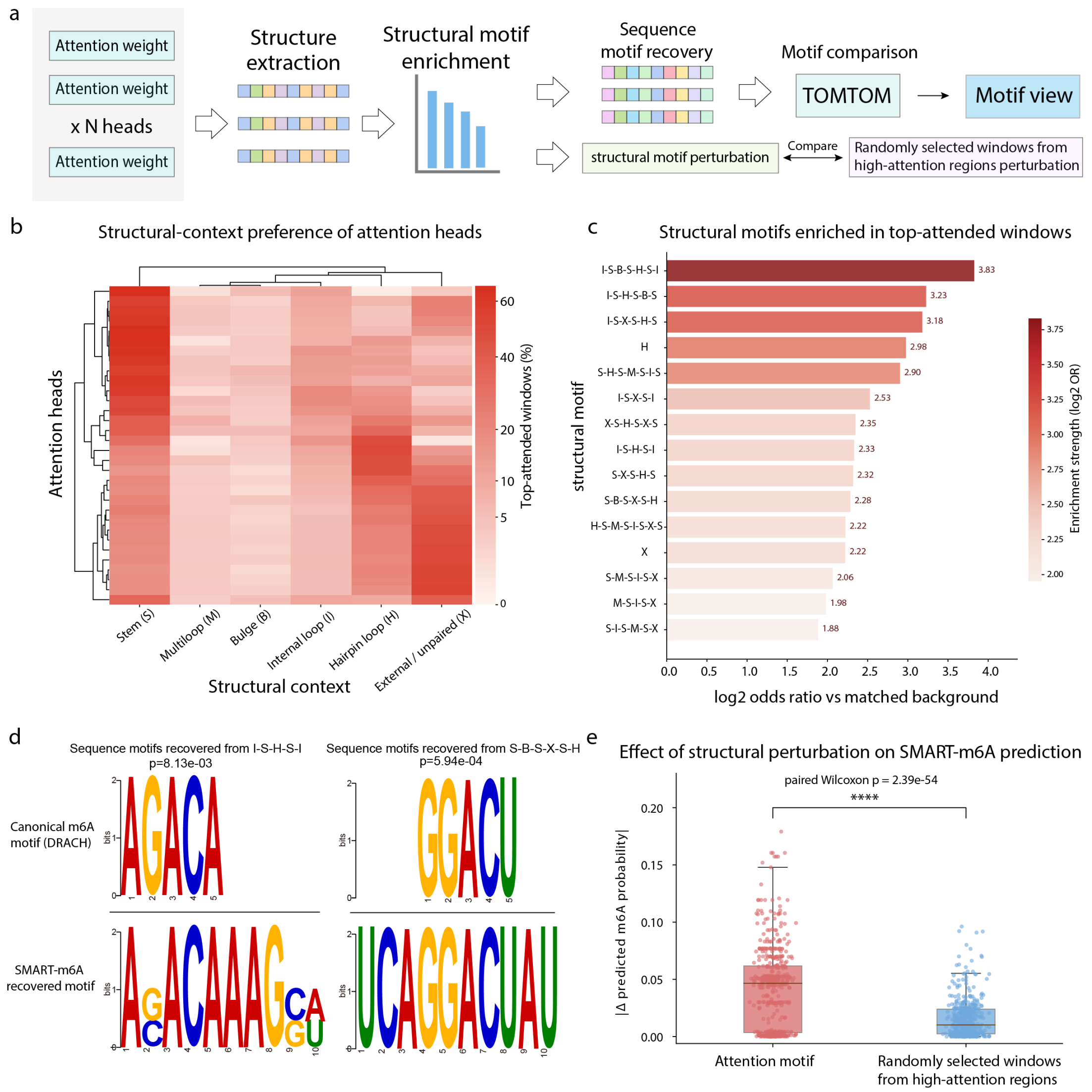

Additional Figure 4. (a) Workflow of attention-guided structural motif analysis. (b) Distribution of top-attended regions across RNA structural contexts for different attention heads. (c) Structural motifs enriched in top-attended windows compared with matched background windows, shown as log2 odds ratios. (d) Recovered sequence motifs from enriched structural motifs matched DRACH-family m6A motifs. (e) Perturbation of attention-selected structural motifs causes larger prediction changes than matched high-attention random perturbations.

We thank the reviewer for this suggestion. We agree that, given the structure-aware design of SMART-m6A, attention-based interpretation should not be limited to sequence motifs alone. In the revised manuscript, we have therefore extended the attention analysis to RNA structural contexts and structural motifs (Additional Figure 4a). Specifically, we mapped

the top-attended regions from each attention head to their corresponding RNA structural contexts, including stem, multiloop, bulge, internal loop, hairpin loop, and exterior/unpaired regions. This analysis is presented in Figure 5e (Additional Figure 4b) and described in Lines 307–311. The results showed that different attention heads exhibited distinct structural-context preferences rather than uniformly attending to all structural categories. For example, some heads preferentially focused on stem-associated regions, whereas others showed stronger attention toward loop or unpaired contexts. These results indicate that the attention mechanism captures heterogeneous structure-associated information across heads.

We further extracted structural motif patterns from the top-attended windows and compared their enrichment against matched background windows. Several structural motifs were enriched in high-attention regions, indicating that the model captures recurrent structural patterns beyond nucleotide sequence motifs. These enriched motifs indicate that local combinations of paired and unpaired structural elements, such as stem-loop, bulge-associated, or junction-like contexts, may help define structural environments favorable for m6A-associated recognition. Thus, these motifs may serve as candidate structure-associated signatures for future experimental validation (Additional Figure 4c).

To further interpret the attention-enriched structural motifs, sequence motifs were recovered from the same windows and were found to match DRACH-family m6A motifs, including GGACA-like and GGACU-like patterns (Additional Figure 4d). These results indicate that the enriched structural motifs occur in sequence contexts consistent with known m6A recognition signals. A matched structural perturbation analysis further showed that perturbing attention-selected structural windows caused larger prediction changes than perturbing high-attention random windows (Additional Figure 4e; Lines 312-318), supporting that these attention-highlighted regions capture coordinated sequence-structure features relevant to SMART-m6A prediction.

Together, these analyses suggest that SMART-m6A attention weights capture multiple layers of sequence-structure information: structural-context preferences across heads, enriched local structural motifs, DRACH-family sequence motifs recovered from these structural-motif windows, and prediction-relevant structural regions validated by perturbation analysis. We have revised the manuscript accordingly by adding this structural attention analysis and by clarifying that these motifs provide computationally derived, biologically interpretable candidates for future mechanistic testing.

Minor Comments

1. Throughout the "Results" section, there are instances of highly speculative language. For example: "These findings highlight the strong biological interpretability and insight offered by a model that integrates structural features, which may further assist in elucidating disease-related epitranscriptomic regulation and in inspiring future therapeutic strategies." The Results section should strictly contain factual descriptions of the data and experimental outcomes. Speculative interpretations and future perspectives should be moved to the "Discussion" section. Please review the entire manuscript to ensure a clear

boundary between results and interpretation.

We thank the reviewer for this suggestion. We have refined the Results section to make the presentation more focused on experimental observations and data-supported findings. In particular, we revised the concluding paragraph of the Figure 4 results. The revised text now reads: “Taken together, SMART-m6A captures structure-associated differences in cell line-specific m6A sites and extends to quantitative prediction of modification intensity across cellular contexts. These results highlight the value of integrating structural features for improving model interpretability and for identifying m6A candidates linked to distinct local structural contexts and cell type-dependent modification patterns.” (Lines 280–284). We also reviewed the Results section more broadly and moved broader biological implications and future perspectives to the Discussion section (Lines 333–362). This revision makes the Results section more focused and keeps the interpretation in the appropriate part of the manuscript.

2. In the section detailing local attention visualizations, the text states: "we zoomed in on a specific head (e.g., head 30) across the first 21 samples." Please clarify the rationale behind selecting exactly "the first 21 samples." Was this an arbitrary cutoff, or is there a statistical or biological justification for this specific subset?

We thank the reviewer for raising this point. The “first 21 samples” were used for local visualization, with samples indexed from 0 to 20 to provide a readable illustration of sample-to-sample heterogeneity in attention patterns for a selected attention head. This displayed range was not intended as a statistically or biologically defined subset. To make this rationale clearer, we revised the manuscript text to describe the visualization as using “a representative attention head and representative positive samples to illustrate sample-to-sample heterogeneity in attention patterns” (Lines 291–293). We also clarified the global attention analysis by showing attention distributions across representative heads in Figure 5b, with additional heads provided in Figure S5 (Lines 284–286).

3. The "Methods" section describes a large number of datasets, making it difficult for the reader to keep track of the data sources, sizes, and specific applications within the study. Please summarize all the datasets used in a comprehensive table to improve clarity and readability.

We thank the reviewer for this suggestion. To make the dataset organization clearer, we added a comprehensive dataset summary table as Table S1 in the Supplementary Information. Table S1 summarizes the dataset names, sources, detection technologies, training and test sizes, and availability of positional information. We also revised the Materials and Methods section to guide readers to this table, with the added sentence: “The dataset sources, detection technologies, training and test sizes, and availability of positional information are summarized in Table S1” (Lines 359–363). With this revision, the main text focuses on the dataset construction strategy, while the detailed dataset information is presented in a structured table for easier reference.

4. Figure 4 presents Pearson correlation coefficients. To increase the statistical rigor of these findings, please include the corresponding p-values for these correlations in the figure or the figure legend.

We thank the reviewer for this suggestion. We revised Figure 4f and Figure 4g to include the corresponding statistical information for the Pearson correlation analyses.

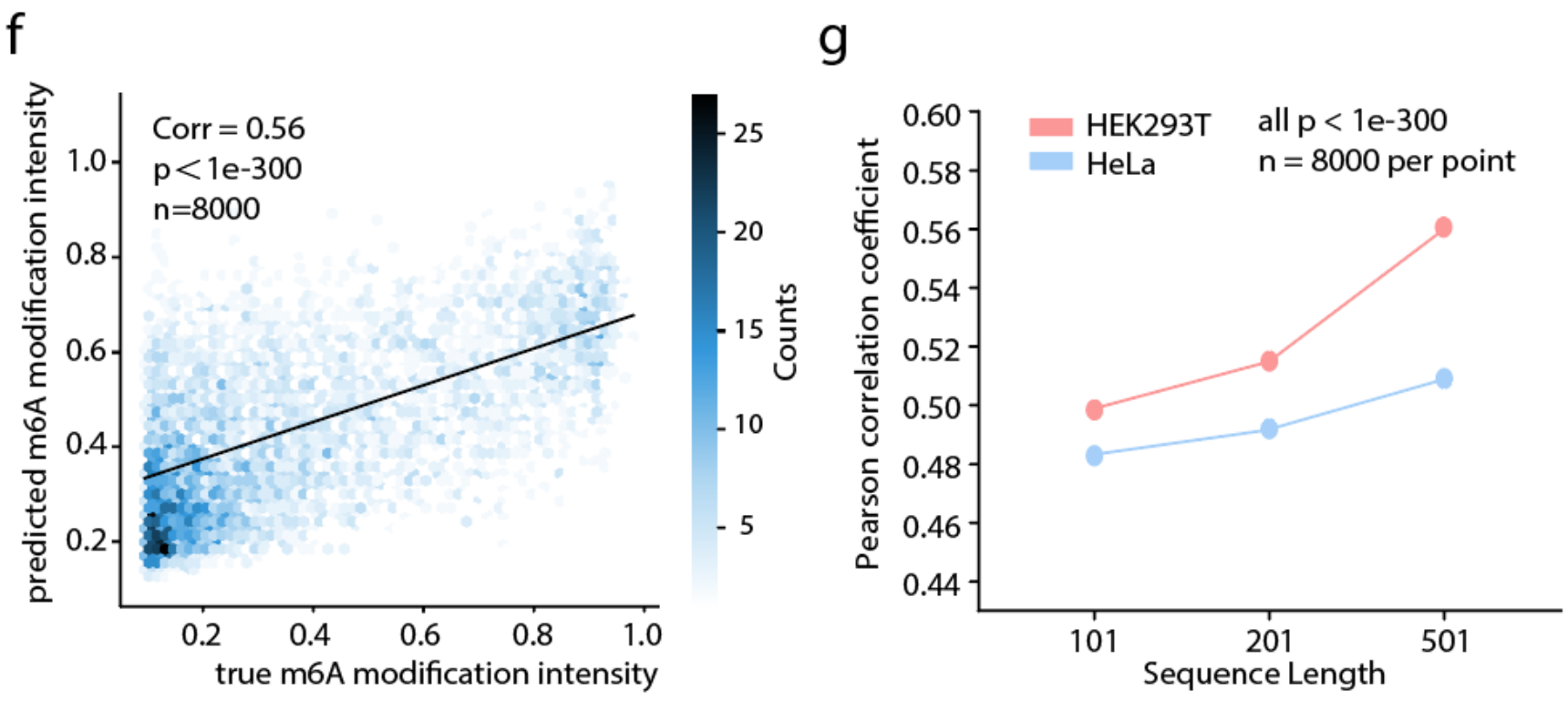

In Figure 4f, we now report the Pearson correlation coefficient together with the two-sided Pearson correlation p-value and sample size directly on the scatter plot. In Figure 4g, because all Pearson correlations across the two cell lines and three sequence lengths were calculated using 8,000 paired samples per point and all tests reached $p < 1e\text{-}300$, we added this information directly to the panel. In the revised Results section, we now state that each correlation was calculated using 8,000 paired samples and that all two-sided Pearson correlation tests were highly significant, with $p < 1e\text{-}300$ (Lines 270–272).

We also added a new Methods subsection entitled “Quantitative regression of m6A modification intensity.” This subsection describes how the regression task was performed and states that predictive performance was evaluated using Pearson correlation coefficients together with corresponding p-values and sample sizes (Lines 479–491). These additions make the statistical support for the regression results more transparent.

5. There are minor typographical issues present in the manuscript. For instance, on line 221 ("m6A deposition, we hypothesized that cell line-specific m6A sites should be associated with more"), there appears to be an incomplete thought or an errant dash. Please thoroughly proofread the manuscript to correct this formatting issue and ensure typographical accuracy throughout.

We thank the reviewer for the careful reading and for pointing out this potential formatting issue. We rechecked the sentence mentioned by the reviewer and revised the surrounding text to remove the ambiguous formatting. The revised sentence now states that RNA secondary structures “undergo dynamic changes across cellular contexts and are associated with m6A-related regulatory features,” followed by the hypothesis that cell line-specific m6A sites may show more pronounced local structural differences (Lines 235–

238). This revision removes the incomplete or ambiguous phrasing in the original manuscript.

We also proofread the entire manuscript to correct typographical, formatting, and punctuation issues, including inconsistent dash usage, spacing around figure and table citations, and minor wording problems. In addition, we corrected terminology inconsistencies such as “multimodal” versus “multifeature”. We believe these revisions have improved the clarity and typographical accuracy of the manuscript.

Reviewer #2:

The authors present SMART-m6A, a multimodal transformer-based framework designed to predict m6A modification sites by integrating RNA primary sequence with predicted secondary structure features. The work is technically sophisticated, utilizing parallel 1D/2D convolutional branches and structure-guided attention. The model demonstrates superior performance over state-of-the-art methods (e.g., deepSRAMP) across multiple benchmark datasets, particularly in scenarios where genomic positional information is unavailable. This "sequence-only" capability is a significant potential benefit, as it allows the tool to be applied to novel transcripts or species without the need for high-quality genomic alignments or positional annotations.
However, while the computational tool is high-performing, the biological interpretation of why it works is subject to several fundamental concerns. There is a significant disconnect between the in vivo biological process of m6A deposition and the in silico modeling of mature mRNA fragments. The authors’ claims regarding structural determinants and writer accessibility are not sufficiently supported by the proposed methodology and may mistake an algorithmic inductive bias for a biological discovery.

We thank the reviewer for the thoughtful and technically insightful assessment of our work. We appreciate the reviewer’s recognition of the technical design of SMART-m6A, including the parallel 1D/2D convolutional branches, structure-guided attention, and its advantage in settings where genomic positional information is unavailable.

In response to the reviewer’s concern, we substantially revised the manuscript to more clearly define the scope of SMART-m6A. The structural features used in our model are now described as sequence-derived structural proxies computed from mature mRNA fragments, rather than as direct representations of native, protein-bound, co-transcriptional RNA structures encountered by the m6A writer complex in vivo. Accordingly, we removed or revised language that could imply causal structural determination of m6A deposition or direct modeling of writer accessibility, including terms such as “structural determinants,” “writer accessibility,” and “guide deposition.”

At the same time, we clarify that this limitation does not mean structural information is dispensable. Although our framework is not designed to establish causality between RNA structure and m6A deposition, the incorporation of structure-derived features provides measurable predictive value. This is supported by ablation analyses, where removing

structural inputs consistently reduced performance, and by hard-sample analysis, where structural information contributed more strongly to ambiguity-prone candidates that were difficult to classify using sequence features alone.

Therefore, we have reframed the contribution of SMART-m6A more conservatively: the model does not prove that RNA secondary structure causally determines m6A deposition, but demonstrates that sequence-derived structural information provides a useful inductive bias and complementary signal for m6A prediction. We also added an explicit limitation in the Discussion to state that SMART-m6A does not reconstruct the native structural environment of m6A deposition, but learns local sequence–structure-associated features that improve prediction and may generate hypotheses for future experimental validation.

Major Concerns:

1. The authors claim that SMART-m6A captures the "structural determinants" guiding m6A deposition by the MTC. However, there is a major disconnect between the biological reality and the computational input: m6A is deposited co-transcriptionally on full-length, protein-bound pre-mRNA in the nucleus. The "writer" complex reacts to the structural landscape of the nascent transcript, which includes introns and long-range interactions. But the model evaluates static, computed secondary structures (via ViennaRNA) on isolated 101-nt fragments of mature mRNA. Splicing and fragmentation destroy the native structural context. A 101-nt fragment folded in silico is heavily biased by artificial fragment ends and does not represent the conformational state "seen" by the writer complex. The authors must revise their manuscript to acknowledge that their model learns a local sequence-structure proxy on mature mRNA, which cannot be equated to in vivo writer accessibility. (And modification aware structure prediction model, like one feature of ViennaRNA model, is not used in this study for cross-validation.)

We thank the reviewer for raising this concern. We agree that SMART-m6A currently uses local mature mRNA fragments as input and therefore cannot represent the complete structural landscape encountered by the m6A writer complex in vivo. Accordingly, we have revised the manuscript to define the folded fragment-level structural features as local sequence-structure proxies, rather than as native MTC-accessible RNA structures or direct indicators of writer accessibility. We have also clarified in the Methods section that all structural features were computed from isolated mature mRNA fragments and that no modification-aware or m6A-aware folding parameters were used during model training or cross-validation. Although such local proxies cannot reconstruct the full in vivo RNA structural environment, they can still provide useful predictive information. The sequence and structural tendency surrounding candidate adenosines may contain signals associated with m6A occurrence, even when they are derived from local mature mRNA fragments. Therefore, SMART-m6A should be interpreted as a structure-aware predictive framework that learns local sequence-structure associated signals, rather than as a direct mechanistic model of co-transcriptional methylation deposition.

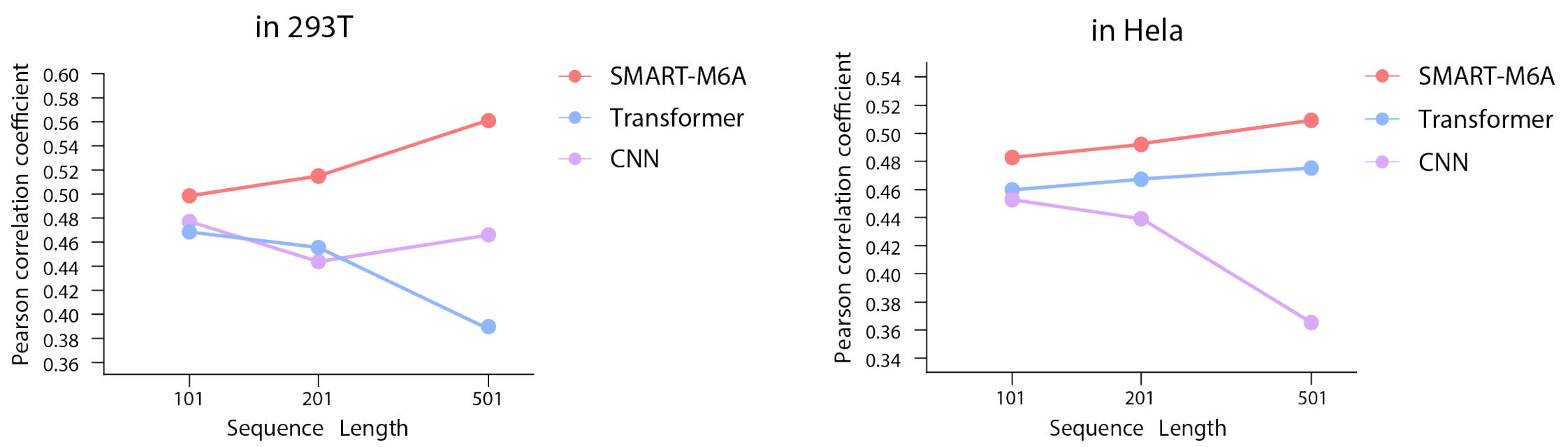

Figure S4 Regression performance of SMART-m6A, Transformer, and CNN at different sequence lengths in 293T and HeLa cell lines.

To further evaluate the usefulness of this local sequence-structure proxy and address the concern that a 101-nt window may be too short, we added an analysis using 101-nt, 201-nt, and 501-nt input windows. As shown in Figure S4, SMART-m6A achieved improved regression performance as the input window increased, whereas the sequence-only Transformer and CNN baselines did not show the same consistent scaling trend. These results suggest that SMART-m6A is not restricted to the 101-nt setting and can benefit from broader flanking sequence-derived structural context when longer local windows are provided. We also emphasize that the 101-nt local-window setting is widely used in m6A prediction studies and is not a limitation introduced only by SMART-m6A. Many existing m6A predictors are also trained and evaluated on short fragments centered on candidate adenosines, because the available benchmark datasets are commonly organized in this way. Within this commonly used local-window setting, the advantage of SMART-m6A is that it attempts to extract additional predictive information from the available fragment-level input by incorporating sequence-derived structural proxies, rather than relying on sequence information alone. This structure-aware design may provide more biologically relevant and interpretable clues about sequence-structure associated methylation patterns. Nevertheless, we interpret Figure S4 cautiously: even a 501-nt window still does not represent a full-length nascent transcript or recover missing intronic sequence, RBP occupancy, long-range RNA interactions, and co-transcriptional folding dynamics. Therefore, Figure S4 supports SMART-m6A as a structure-aware local-context model with the capacity to use broader local context, rather than as evidence that the model reconstructs the complete in vivo structural landscape encountered by the m6A writer complex.

2. The authors use icSHAPE data (Line 226) to validate that structural sensitivity determines m6A sites. This introduces a significant logical risk. Extensive literature confirms that the presence of m6A itself destabilizes RNA duplexes and alters the local secondary structure (the m6A-switch). If the icSHAPE data was derived from cells where m6A was already present (and I think most it is most likely), the "structural sensitivity" observed might be the result of methylation rather than the cause. Without performing METTL3-knockout experiments to verify that these structures exist independently of the modification, the claim that "structure determines m6A" is potentially just circular.

We thank the reviewer for raising this point. We agree that icSHAPE profiles measured from cellular RNA cannot establish the causal direction between RNA structure and m6A modification. As the reviewer noted, m6A can destabilize local RNA duplexes and induce structural remodeling through the m6A-switch mechanism. Therefore, structural differences observed in icSHAPE data may reflect pre-existing structural features associated with m6A deposition, m6A-induced structural remodeling, or both.

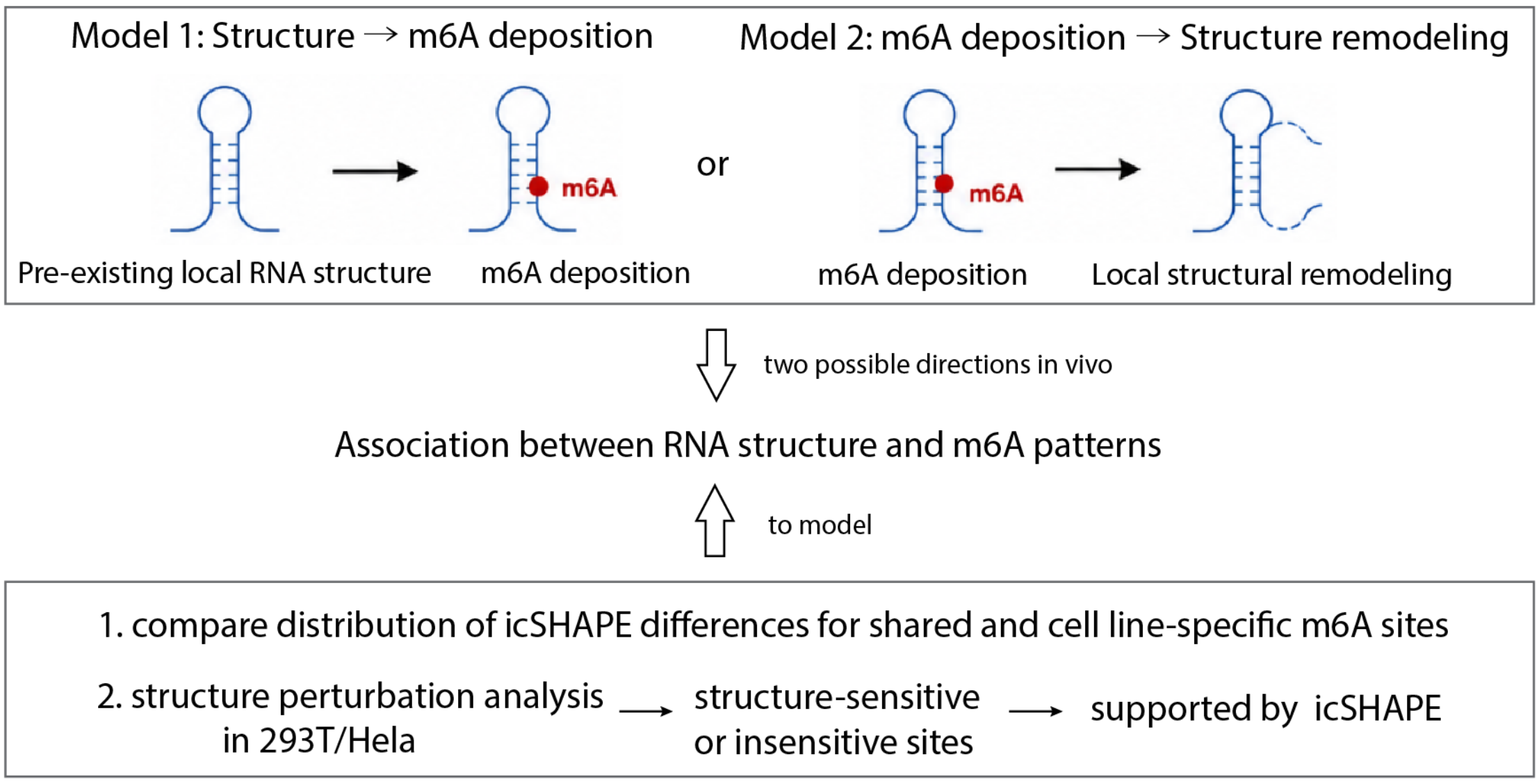

Additional Figure 5. Schematic overview of two possible in vivo relationships between local RNA structure and m6A modification: structure-associated m6A deposition and m6A-associated structural remodeling. The lower workflow outlines the analyses used to model the association between RNA structure and m6A patterns by comparing icSHAPE divergence and classifying sites as structure-sensitive or structure-insensitive through structure perturbation.

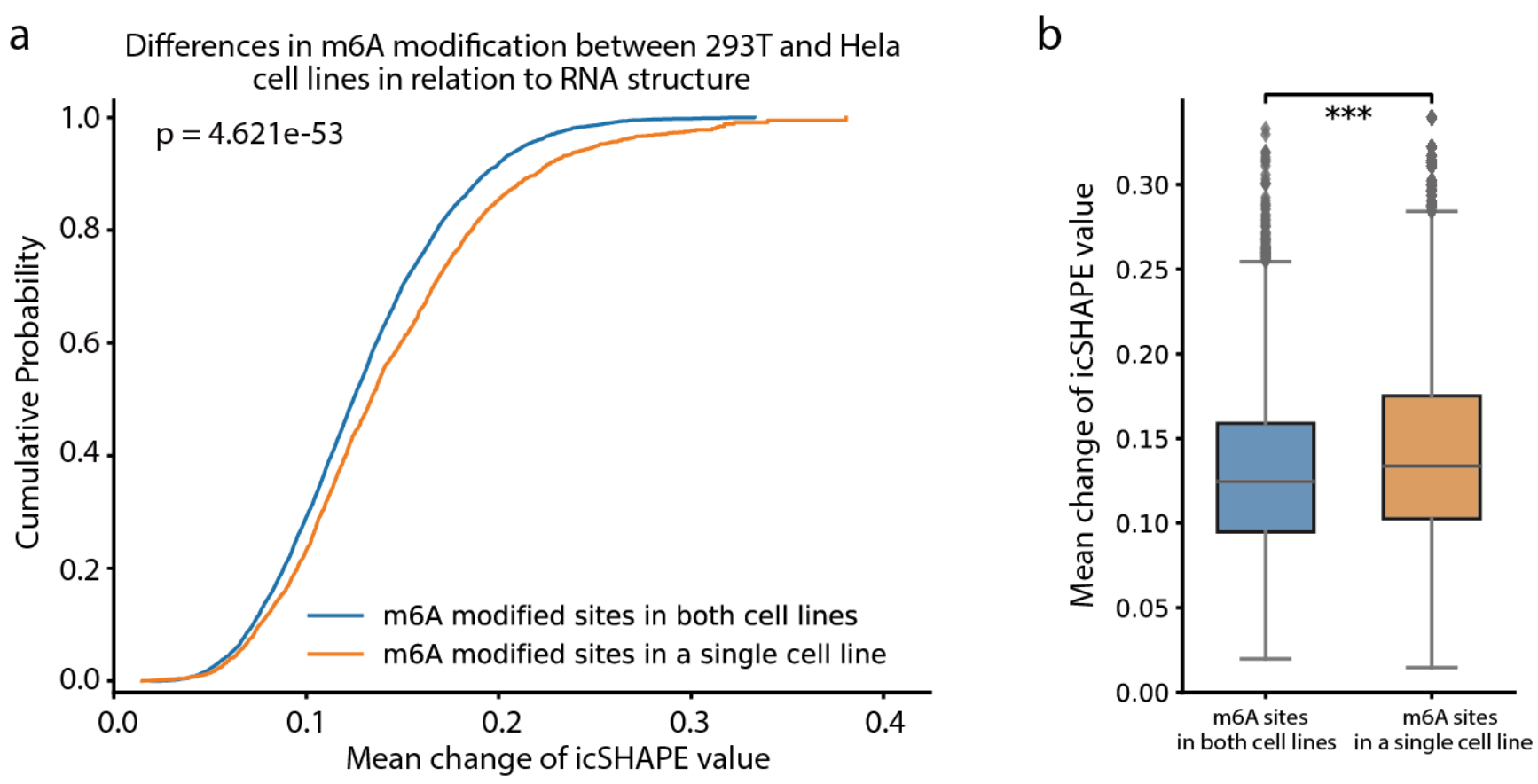

Additional Figure 6. (a) Cumulative distribution of icSHAPE differences for shared and cell line-specific m6A sites. (b) Boxplot comparison of icSHAPE differences between shared and cell line-specific m6A sites.

To address this concern, we have revised the manuscript to remove causal wording from the interpretation of the icSHAPE analysis. In the revised manuscript, RNA structure is described in terms of structure-associated differences, and icSHAPE analysis is

interpreted as evidence of association, without implying causal validation. Instead, we now describe these results in terms of association, concordance, and structure-associated differences. We illustrated Additional Figure 5 to explicitly clarify this causal boundary. This schematic illustrates two possible in vivo relationships between local RNA structure and m6A modification: structure-associated m6A deposition and m6A-associated structural remodeling. The lower workflow in Additional Figure 5 further clarifies that our analyses were designed to model the association between RNA structure and m6A patterns, rather than to infer a unidirectional causal mechanism.

In this revised framework, icSHAPE is used as an independent in vivo structural readout, not as evidence that RNA structure causes m6A deposition. Additional Figure 6 (Figure 4b) shows that cell line-specific m6A sites have larger local icSHAPE differences between HEK293T and HeLa than shared m6A sites, supporting an association between cell type-dependent m6A patterns and local RNA structural variation. The role of the structure perturbation analysis was also clarified. This model-based assay defines SMART-m6A structure-sensitive sites according to prediction changes after local structural perturbation, and the subsequent icSHAPE comparison examines whether these sites show concordant experimental structural divergence (Figure 4d). Thus, these analyses assess whether SMART-m6A captures structure-associated m6A patterns, without assigning causal direction between RNA structure and m6A modification.

3. While the model shows improved accuracy, the authors must clarify why. Because the secondary structure features (BPP matrices) are deterministically calculated from the 101-nt sequence letters, no novel in vivo biological information is actually being added to the system. It is highly likely that the structure module acts as a structural regularizer or inductive bias. By forcing the network to attend to base-pairing probabilities, the framework likely reduces native sequence noise, allowing the model to optimize faster. The model is likely not learning that METTL3 cannot reach this site, but rather that mathematically grouping complementary letters improves classification scores. The authors should tone down mechanistic claims of such "accessibility" and address this computational interpretation.

We thank the reviewer for this comment. We agree that the structural features used in SMART-m6A are deterministically derived from the 101-nt input sequence and therefore do not introduce independent in vivo structural measurements. These features should not be interpreted as direct readouts of methyltransferase accessibility. Instead, they provide a folding-informed reparameterization of the input sequence. Under this interpretation, the structural branch introduces RNA folding rules and biophysical constraints as explicit prior information, rather than requiring the neural network to infer all higher-order base-pairing relationships directly from raw nucleotide letters. Thus, the contribution of structure-derived features is best understood as a computationally useful biological/biophysical inductive bias: they make local structural tendencies explicit to the model, while remaining sequence-derived local structural proxies. Accordingly, we have toned down mechanistic statements related to writer accessibility, structural determinants, and causal m6A deposition in the revised manuscript, and now describe the structural inputs as sequence-

derived local structural representations that support predictive sequence–structure-associated modeling.

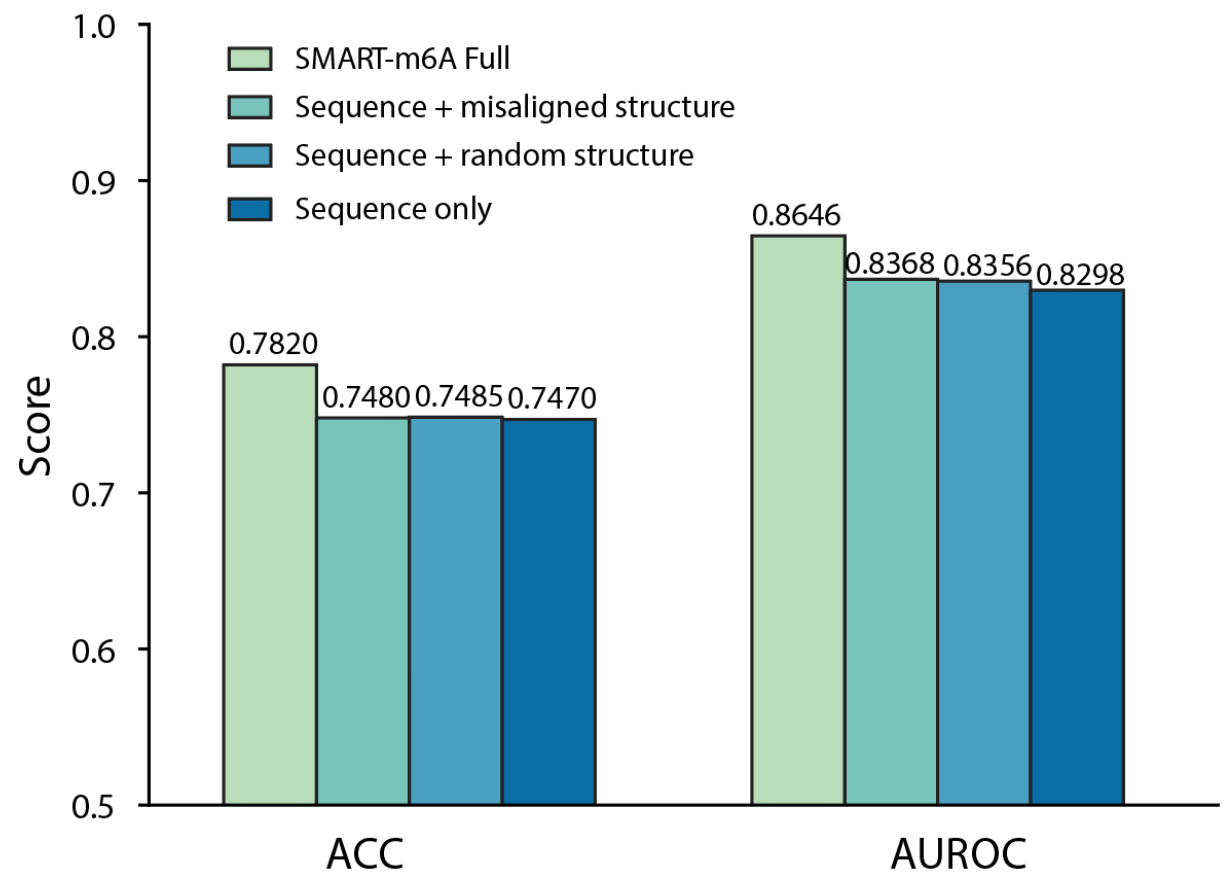

Additional Figure 7. Comparison of ACC and AUROC among SMART-m6A Full, sequence + misaligned structure, sequence + randomly shuffled structure, and sequence-only SMART-m6A on the SMART-m6A dataset.

To further address whether the observed improvement simply reflects increased model capacity, arbitrary regularization, or the presence of an additional structural branch, we added structure-misalignment and random-structure control analyses (Additional Figure 7; Figure S1). In the structure-misalignment control, sequence inputs and labels were kept unchanged, whereas the structural inputs were shuffled across samples, breaking the correct correspondence between each sequence and its derived structural representation. In the random-structure control, structural representations were randomized while preserving the same sequence inputs, labels, structural branch, input format, and model parameterization. Both control models performed similarly to the sequence-only model, whereas the full SMART-m6A model achieved higher Accuracy and AUROC. These results indicate that the gain is not explained by arbitrary structural inputs or model expansion alone, but depends on preserving the correct sequence–structure correspondence. We therefore interpret the structural module as a folding-informed biological/biophysical prior that improves prediction by organizing sequence information under RNA structural constraints, rather than as direct evidence that SMART-m6A has learned in vivo METTL3/METTL14 accessibility or established a causal structural mechanism of m6A deposition.

4. In the Results section (Fig 4e-g), the authors highlight the model's ability to predict modification intensity (stoichiometry). However, the Methods section (Lines 427-450) only describes a binary classification framework (MLP with Cross-Entropy loss). I think the authors have omitted the methodology for the regression model. It is unclear what loss function was used (e.g., MSE, MAE), how the modification intensity was normalized, or how the architecture was adapted for a quantitative output.

We thank the reviewer for pointing out this omission. To address this issue, we added a new subsection in the Methods section entitled "Quantitative regression of m6A

modification intensity" (Lines 479–491). In this subsection, we clarified how SMART-m6A was adapted from binary classification to regression. Specifically, the StructureTransformer encoder was kept unchanged, while the final classification layer was replaced with a single-neuron regression head. GLORI-derived m6A modification levels were used as continuous quantitative labels and were represented on a normalized scale between 0 and 1. The regression model was optimized using mean squared error loss between predicted and observed modification levels (Lines 479–486).

We also clarified that models were trained separately for HEK293T and HeLa datasets to account for cell line-specific modification profiles, and that 101-nt, 201-nt, and 501-nt input windows were used to evaluate the effect of sequence context length. Predictive performance was evaluated using Pearson correlation coefficients between predicted and observed modification levels, together with corresponding p-values and sample sizes (Lines 487–491).

Minor Concerns:
1. The 101-nt window is a narrow slice of a transcript. Structural determinants often involve nucleotides hundreds of bases away, which are excluded here.

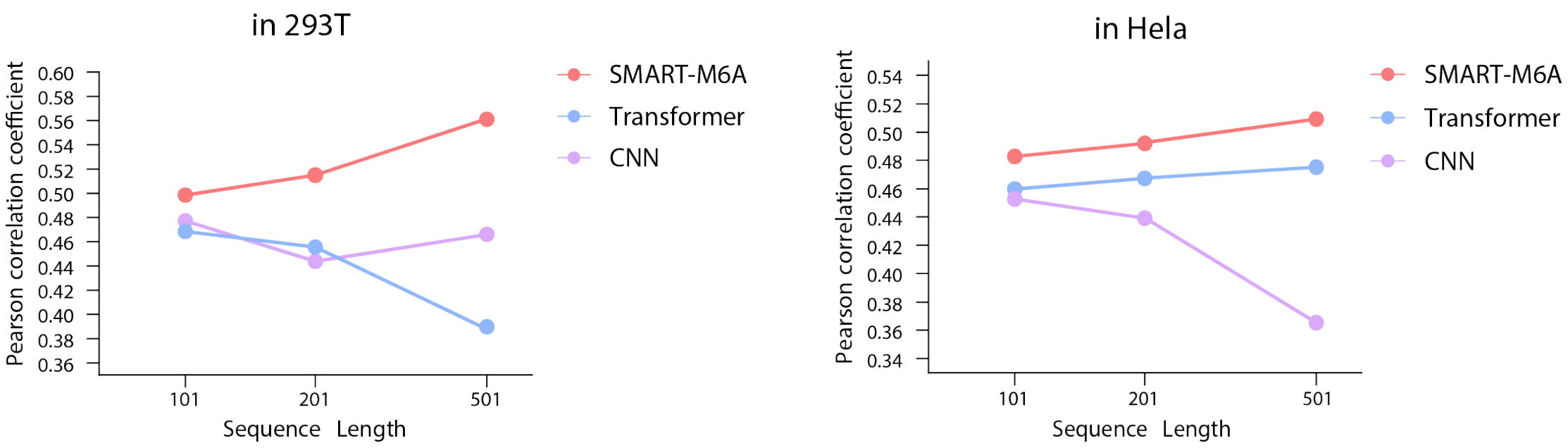

Figure S4 Regression performance of SMART-m6A, Transformer, and CNN at different sequence lengths in 293T and HeLa cell lines.

We thank the reviewer for this comment. We agree that a 101-nt window represents only a local region around the candidate m6A site and cannot capture transcript-level or long-range RNA structural interactions. The reason we used the 101-nt window in the main benchmark is that most existing m6A prediction datasets and baseline methods are constructed using short mRNA fragments centered on candidate adenosines. Using the same setting therefore allows a fair comparison with previous methods under matched input conditions. At the same time, we agree that broader flanking context may contain additional structural information. To address this point, we further provided extended-window versions of the model using 201-nt and 501-nt input sequences, in addition to the original 101-nt setting. This supplementary analysis was designed to examine whether SMART-m6A can benefit from longer sequence-derived structural context when such information is available. As shown in Figure S4, SMART-m6A showed improved regression performance as the input window increased from 101-nt to 201-nt and 501-nt, whereas the sequence-only Transformer and CNN baselines did not show the same consistent scaling trend. These results suggest that the structure-aware design of SMART-m6A can make use of broader flanking context beyond the standard 101-nt benchmark setting. They

demonstrate that SMART-m6A is not restricted to the 101-nt setting and can be extended to incorporate broader local sequence-derived structural context.

2. The term "Multimodal" is usually reserved for distinct data types (e.g., image + text). Since the "Structure" mode is derived directly from the Sequence, multi-feature or structure-integrated might be more accurate.

We thank the reviewer for this suggestion. This suggestion prompted us to improve the precision of our description of SMART-m6A. Accordingly, we replaced “multimodal” with “multifeature” throughout the revised manuscript. Specifically, the model name in the Abstract now reads “Sequence-structure Multifeature Attention RNA Transformer for m6A,” and the fusion process is described as “multifeature fusion” (Lines 32–35). The Author Summary now refers to a “multifeature neural architecture” (Lines 55–57), the Introduction introduces SMART-m6A as a “multifeature deep learning framework” (Line 129), the Discussion uses the same terminology (Line 327), and the Figure 1 caption now describes “multifeature sequence-structure integration” (Line 653). This revised terminology more accurately reflects the structure-integrated design of SMART-m6A as a framework that integrates sequence-derived structural features with nucleotide sequence information.
