# Supplementary material for: Structure-aware deep learning enhances m6A prediction and reveals cell type-associated RNA structural signatures
Source: PLoS Comput Biol. 2026 Aug 14;22(8):e1014649. doi: 10.1371/journal.pcbi.1014649 (PMC13502593; doi:10.1371/journal.pcbi.1014649)
Supplement: Supplementary file 2 [file pcbi.1014649.s010.docx]

**Graphical Abstract**
