# Supplementary material for: Structure-aware deep learning enhances m6A prediction and reveals cell type-associated RNA structural signatures
Source: PLoS Comput Biol. 2026 Aug 14;22(8):e1014649. doi: 10.1371/journal.pcbi.1014649 (PMC13502593; doi:10.1371/journal.pcbi.1014649)
Supplement: Peer review history

# Peer review: Decision Letter 0

**DOI:** 10.1371/journal.pcbi.1014649.r001

**Authors:** Stacey D. Finley, Hatice Ulku Osmanbeyoglu

9 Apr 2026

Structure-aware deep learning enhances m6A prediction and reveals cell type-dependent structural determinants

PLOS Computational Biology

Dear Dr. Lin,

Thank you for submitting your manuscript to PLOS Computational Biology. After careful consideration, we feel that it has merit but does not fully meet PLOS Computational Biology's publication criteria as it currently stands. Therefore, we invite you to submit a revised version of the manuscript that addresses the points raised during the review process.

Please submit your revised manuscript by Jun 09 2026 11:59PM. If you will need more time than this to complete your revisions, please reply to this message or contact the journal office at ploscompbiol@plos.org. When you're ready to submit your revision, log on to https://www.editorialmanager.com/pcompbiol/ and select the 'Submissions Needing Revision' folder to locate your manuscript file.

Please include the following items when submitting your revised manuscript:

* A letter that responds to each point raised by the editor and reviewer(s). You should upload this letter as a separate file labeled 'Response to Reviewers'. This file does not need to include responses to formatting updates and technical items listed in the 'Journal Requirements' section below.

* A marked-up copy of your manuscript that highlights changes made to the original version. You should upload this as a separate file labeled 'Revised Manuscript with Track Changes'.

* An unmarked version of your revised paper without tracked changes. You should upload this as a separate file labeled 'Manuscript'.

If you would like to make changes to your financial disclosure, competing interests statement, or data availability statement, please make these updates within the submission form at the time of resubmission. Guidelines for resubmitting your figure files are available below the reviewer comments at the end of this letter

We look forward to receiving your revised manuscript.

Kind regards,

Hatice Ulku Osmanbeyoglu, Ph.D

Academic Editor

PLOS Computational Biology

Stacey Finley, Ph.D.

Section Editor

PLOS Computational Biology

**Additional Editor Comments:**

The reviewers raise concerns regarding the limitations of the method, performance gain, and biological insights that can be obtained. The Authors are urged to thoroughly address these concerns.

**Journal Requirements:**

If the reviewer comments include a recommendation to cite specific previously published works, please review and evaluate these publications to determine whether they are relevant and should be cited. There is no requirement to cite these works unless the editor has indicated otherwise.

1) Please ensure that the CRediT author contributions listed for every co-author are completed accurately and in full.

At this stage, the following Authors/Authors require contributions: Yihan Lin. Please ensure that the full contributions of each author are acknowledged in the "Add/Edit/Remove Authors" section of our submission form.

The list of CRediT author contributions may be found here: https://journals.plos.org/ploscompbiol/s/authorship#loc-author-contributions

2) We ask that a manuscript source file is provided at Revision. Please upload your manuscript file as a .doc, .docx, .rtf or .tex. If you are providing a .tex file, please upload it under the item type u2018LaTeX Source Fileu2019 and leave your .pdf version as the item type u2018Manuscriptu2019.

3) Please upload all main figures as separate Figure files in .tif or .eps format. For more information about how to convert and format your figure files please see our guidelines:

https://journals.plos.org/ploscompbiol/s/figures

4) We have noticed that you have uploaded Supporting Information files, but you have not included a list of legends. Please add a full list of legends for your Supporting Information files after the references list.

5) Please provide a detailed Financial Disclosure statement. This is published with the article. It must therefore be completed in full sentences and contain the exact wording you wish to be published.

1) Please clarify all sources of financial support for your study. List the grants, grant numbers, and organizations that funded your study, including funding received from your institution. Please note that suppliers of material support, including research materials, should be recognized in the Acknowledgements section rather than in the Financial Disclosure

2) State the initials, alongside each funding source, of each author to receive each grant. For example: "This work was supported by the National Institutes of Health (####### to AM; ###### to CJ) and the National Science Foundation (###### to AM)."

3) State what role the funders took in the study. If the funders had no role in your study, please state: "The funders had no role in study design, data collection and analysis, decision to publish, or preparation of the manuscript."

4) If any authors received a salary from any of your funders, please state which authors and which funders..

If you did not receive any funding for this study, please simply state: u201cThe authors received no specific funding for this work.u201d

6) Your current Financial Disclosure states, "The author(s) received no specific funding for this work.".

However, your funding information on the submission form indicates receiving fund from Sichuan Science and Technology Program.

Please indicate by return email the full and correct funding information for your study and confirm the order in which funding contributions should appear. Please be sure to indicate whether the funders played any role in the study design, data collection and analysis, decision to publish, or preparation of the manuscript.

**Reviewers' comments:**

Reviewer's Responses to Questions

**Comments to the Authors:**

**Please note here if the review is uploaded as an attachment.**

Reviewer #1: This manuscript presents SMART-m6A, a deep learning tool that integrates structural features to predict m6A sites and identify cell-type-specific patterns. The premise of incorporating RNA structure into epitranscriptomic prediction is interesting. The insight into the association of RNA structure with m6A can be useful to future biological research some translational applications, such as mRNA therapy. There are several concerns regarding the justification of the model's complexity relative to its performance gains, as well as the interpretation and presentation of the results, that need to be addressed.

Major Comments

The authors construct a complex Transformer-based model to incorporate structural features; however, the performance improvements appear marginal. In Tables 1-5 and Figures 2c-2d, SMART-m6A improves the AUC over XGBoost by only about 3%, with other metrics showing only slight, and sometimes inconsistent, improvements. Given that such small differences can often be attributed to hyperparameter tuning variations rather than fundamental architectural advantages, the necessity of this complex model is questionable.

The authors state that handcrafted features fail to capture underlying rules due to intricate RNA sequence interactions, yet SMART-m6A does not yield a substantial leap in predictive accuracy.

In the ablation experiments, removing structural information only decreases accuracy by 1.4–4.1% and AUROC by 0.68–3.48%. Does a performance drop of 1-4% truly indicate that structural information plays a significant enough role to be the primary novel contribution of this paper?

The authors claim that cell-type-specific m6A patterns are linked to RNA structure. While this is a compelling hypothesis, it currently lacks concrete visual or data-driven examples in the text. To substantiate this claim, please provide specific examples demonstrating the structural differences at these key m6A sites across different cell types.

The study successfully utilizes the attention mechanism for sequence motif analysis. Given the paper's focus on RNA structure, restricting the attention analysis strictly to sequences feels like a missed opportunity. Could the attention mechanism also be applied to structural analysis? It would significantly strengthen the paper if the authors could demonstrate whether the model's attention weights can identify specific structural motifs or conformations that provide physical evidence for the presence of m6A.

Minor Comments

Throughout the "Results" section, there are instances of highly speculative language. For example: "These findings highlight the strong biological interpretability and insight offered by a model that integrates structural features, which may further assist in elucidating disease-related epitranscriptomic regulation and in inspiring future therapeutic strategies." The Results section should strictly contain factual descriptions of the data and experimental outcomes. Speculative interpretations and future perspectives should be moved to the "Discussion" section. Please review the entire manuscript to ensure a clear boundary between results and interpretation.

In the section detailing local attention visualizations, the text states: "we zoomed in on a specific head (e.g., head 30) across the first 21 samples." Please clarify the rationale behind selecting exactly "the first 21 samples." Was this an arbitrary cutoff, or is there a statistical or biological justification for this specific subset?

The "Methods" section describes a large number of datasets, making it difficult for the reader to keep track of the data sources, sizes, and specific applications within the study. Please summarize all the datasets used in a comprehensive table to improve clarity and readability.

Figure 4 presents Pearson correlation coefficients. To increase the statistical rigor of these findings, please include the corresponding p-values for these correlations in the figure or the figure legend.

There are minor typographical issues present in the manuscript. For instance, on line 221 ("m6A deposition, we hypothesized that cell line-specific m6A sites should be associated with more"), there appears to be an incomplete thought or an errant dash. Please thoroughly proofread the manuscript to correct this formatting issue and ensure typographical accuracy throughout.

Reviewer #2: The authors present SMART-m6A, a multimodal transformer-based framework designed to predict m6A modification sites by integrating RNA primary sequence with predicted secondary structure features. The work is technically sophisticated, utilizing parallel 1D/2D convolutional branches and structure-guided attention. The model demonstrates superior performance over state-of-the-art methods (e.g., deepSRAMP) across multiple benchmark datasets, particularly in scenarios where genomic positional information is unavailable. This "sequence-only" capability is a significant potential benefit, as it allows the tool to be applied to novel transcripts or species without the need for high-quality genomic alignments or positional annotations.

However, while the computational tool is high-performing, the biological interpretation of why it works is subject to several fundamental concerns. There is a significant disconnect between the in vivo biological process of m6A deposition and the in silico modeling of mature mRNA fragments. The authors’ claims regarding structural determinants and writer accessibility are not sufficiently supported by the proposed methodology and may mistake an algorithmic inductive bias for a biological discovery.

Major Concerns:

1. The authors claim that SMART-m6A captures the "structural determinants" guiding m6A deposition by the MTC. However, there is a major disconnect between the biological reality and the computational input: m6A is deposited co-transcriptionally on full-length, protein-bound pre-mRNA in the nucleus. The "writer" complex reacts to the structural landscape of the nascent transcript, which includes introns and long-range interactions. But the model evaluates static, computed secondary structures (via ViennaRNA) on isolated 101-nt fragments of mature mRNA. Splicing and fragmentation destroy the native structural context. A 101-nt fragment folded in silico is heavily biased by artificial fragment ends and does not represent the conformational state "seen" by the writer complex. The authors must revise their manuscript to acknowledge that their model learns a local sequence-structure proxy on mature mRNA, which cannot be equated to in vivo writer accessibility. (And modification aware structure prediction model, like one feature of ViennaRNA model, is not used in this study for cross-validation.)

2. The authors use icSHAPE data (Line 226) to validate that structural sensitivity determines m6A sites. This introduces a significant logical risk. Extensive literature confirms that the presence of m6A itself destabilizes RNA duplexes and alters the local secondary structure (the m6A-switch). If the icSHAPE data was derived from cells where m6A was already present (and I think most it is most likely), the "structural sensitivity" observed might be the result of methylation rather than the cause. Without performing METTL3-knockout experiments to verify that these structures exist independently of the modification, the claim that "structure determines m6A" is potentially just circular.

3. While the model shows improved accuracy, the authors must clarify why. Because the secondary structure features (BPP matrices) are deterministically calculated from the 101-nt sequence letters, no novel in vivo biological information is actually being added to the system. It is highly likely that the structure module acts as a structural regularizer or inductive bias. By forcing the network to attend to base-pairing probabilities, the framework likely reduces native sequence noise, allowing the model to optimize faster. The model is likely not learning that METTL3 cannot reach this site, but rather that mathematically grouping complementary letters improves classification scores. The authors should tone down mechanistic claims of such "accessibility" and address this computational interpretation.

4. In the Results section (Fig 4e-g), the authors highlight the model’s ability to predict modification intensity (stoichiometry). However, the Methods section (Lines 427-450) only describes a binary classification framework (MLP with Cross-Entropy loss). I think the authors have omitted the methodology for the regression model. It is unclear what loss function was used (e.g., MSE, MAE), how the modification intensity was normalized, or how the architecture was adapted for a quantitative output.

Minor Concerns:

1. The 101-nt window is a narrow slice of a transcript. Structural determinants often involve nucleotides hundreds of bases away, which are excluded here.

2. The term "Multimodal" is usually reserved for distinct data types (e.g., image + text). Since the "Structure" mode is derived directly from the Sequence, multi-feature or structure-integrated might be more accurate.

**********

**Have the authors made all data and (if applicable) computational code underlying the findings in their manuscript fully available?**

The PLOS Data policy requires authors to make all data and code underlying the findings described in their manuscript fully available without restriction, with rare exception (please refer to the Data Availability Statement in the manuscript PDF file). The data and code should be provided as part of the manuscript or its supporting information, or deposited to a public repository. For example, in addition to summary statistics, the data points behind means, medians and variance measures should be available. If there are restrictions on publicly sharing data or code —e.g. participant privacy or use of data from a third party—those must be specified.

Reviewer #1: Yes

Reviewer #2: **No:**  chech the comments to authors

**********

PLOS authors have the option to publish the peer review history of their article (what does this mean?). If published, this will include your full peer review and any attached files.

If you choose “no”, your identity will remain anonymous but your review may still be made public.

**Do you want your identity to be public for this peer review?**  For information about this choice, including consent withdrawal, please see our Privacy Policy.

Reviewer #1: No

Reviewer #2: No

**Figure resubmission:**

After uploading your figures to PLOS’s NAAS tool - https://ngplosjournals.pagemajik.ai/artanalysis, NAAS will process the files provided and display the results in the "Uploaded Files" section of the page as the processing is complete. If the uploaded figures meet our requirements (or NAAS is able to fix the files to meet our requirements), the figure will be marked as "fixed" above. If NAAS is unable to fix the files, a red "failed" label will appear above. When NAAS has confirmed that the figure files meet our requirements, please download the file via the download option, and include these NAAS processed figure files when submitting your revised manuscript.

**Reproducibility:**

To enhance the reproducibility of your results, we recommend that authors of applicable studies deposit laboratory protocols in protocols.io, where a protocol can be assigned its own identifier (DOI) such that it can be cited independently in the future. Additionally, PLOS ONE offers an option to publish peer-reviewed clinical study protocols. Read more information on sharing protocols at https://plos.org/protocols?utm_medium=editorial-email&utm_source=authorletters&utm_campaign=protocols

# Author comment: Author response to Decision Letter 1

**DOI:** 10.1371/journal.pcbi.1014649.r002

9 Jun 2026

Attachment Submitted filename: Response to Reviewers.pdf

# Peer review: Decision Letter 1

**DOI:** 10.1371/journal.pcbi.1014649.r003

**Authors:** Marc R Birtwistle

2 Aug 2026

Dear Dr. Lin,

We are pleased to inform you that your manuscript 'Structure-aware deep learning enhances m6A prediction and reveals cell type-associated RNA structural signatures' has been provisionally accepted for publication in PLOS Computational Biology.

Before your manuscript can be formally accepted you will need to complete some formatting changes, which you will receive in a follow up email. A member of our team will be in touch with a set of requests.

Please note that your manuscript will not be scheduled for publication until you have made the required changes, so a swift response is appreciated.

IMPORTANT: The editorial review process is now complete. PLOS will only permit corrections to spelling, formatting or significant scientific errors from this point onwards. Requests for major changes, or any which affect the scientific understanding of your work, will cause delays to the publication date of your manuscript.

Should you, your institution's press office or the journal office choose to press release your paper, you will automatically be opted out of early publication. We ask that you notify us now if you or your institution is planning to press release the article. All press must be co-ordinated with PLOS.

Thank you again for supporting Open Access publishing; we are looking forward to publishing your work in PLOS Computational Biology.

Best regards,

Marc Birtwistle, PhD

Section Editor

PLOS Computational Biology

Stacey Finley

Section Editor

PLOS Computational Biology

***********************************************************

Reviewer's Responses to Questions

**Comments to the Authors:**

**Please note here if the review is uploaded as an attachment.**

Reviewer #1: My comments have been well addressed in the revised manuscript.

**********

**Have the authors made all data and (if applicable) computational code underlying the findings in their manuscript fully available?**

The PLOS Data policy requires authors to make all data and code underlying the findings described in their manuscript fully available without restriction, with rare exception (please refer to the Data Availability Statement in the manuscript PDF file). The data and code should be provided as part of the manuscript or its supporting information, or deposited to a public repository. For example, in addition to summary statistics, the data points behind means, medians and variance measures should be available. If there are restrictions on publicly sharing data or code —e.g. participant privacy or use of data from a third party—those must be specified.

Reviewer #1: Yes

**********

PLOS authors have the option to publish the peer review history of their article (what does this mean?). If published, this will include your full peer review and any attached files.

If you choose “no”, your identity will remain anonymous but your review may still be made public.

**Do you want your identity to be public for this peer review?**  For information about this choice, including consent withdrawal, please see our Privacy Policy.

Reviewer #1: No

# Editor report: Acceptance letter

**DOI:** 10.1371/journal.pcbi.1014649.r004

**Authors:** Marc R Birtwistle

PCOMPBIOL-D-26-00544R1

Structure-aware deep learning enhances m6A prediction and reveals cell type-associated RNA structural signatures

Dear Dr Lin,

I am pleased to inform you that your manuscript has been formally accepted for publication in PLOS Computational Biology. Your manuscript is now with our production department and you will be notified of the publication date in due course.

The corresponding author will soon be receiving a typeset proof for review, to ensure errors have not been introduced during production. Please review the PDF proof of your manuscript carefully, as this is the last chance to correct any errors. Please note that major changes, or those which affect the scientific understanding of the work, will likely cause delays to the publication date of your manuscript.

Soon after your final files are uploaded, unless you have opted out, the early version of your manuscript will be published online. The date of the early version will be your article's publication date. The final article will be published to the same URL, and all versions of the paper will be accessible to readers.

For Research, Software, and Methods articles, you will receive an invoice from PLOS for your publication fee after your manuscript has reached the completed accept phase. If you receive an email requesting payment before acceptance or for any other service, this may be a phishing scheme. Learn how to identify phishing emails and protect your accounts at https://explore.plos.org/phishing.

Thank you again for supporting PLOS Computational Biology and open-access publishing. We are looking forward to publishing your work!

With kind regards,

Kannan R K Kuppusamy, B.TECH BIOTECHNOLOGY

PLOS Computational Biology | Carlyle House, Carlyle Road, Cambridge CB4 3DN | United Kingdom ploscompbiol@plos.org | Phone +44 (0) 1223-442824 | ploscompbiol.org | @PLOSCompBiol
